# Evaluating the Potential of Machine Learning and Wearable Devices in End-of-Life Care in Predicting 7-Day Death Events Among Patients With Terminal Cancer: Cohort Study

**DOI:** 10.2196/47366

**Published:** 2023-08-18

**Authors:** Jen-Hsuan Liu, Chih-Yuan Shih, Hsien-Liang Huang, Jen-Kuei Peng, Shao-Yi Cheng, Jaw-Shiun Tsai, Feipei Lai

**Affiliations:** 1 Department of Family Medicine National Taiwan University Hospital Hsin-Chu Branch Hsin-Chu Taiwan; 2 Graduate Institute of Biomedical Electronics and Bioinformatics National Taiwan University Taipei Taiwan; 3 Department of Family Medicine National Taiwan University Hospital National Taiwan University Taipei Taiwan; 4 Department of Family Medicine College of Medicine National Taiwan University Taipei Taiwan; 5 Department of Computer Science and Information Engineering National Taiwan University Taipei Taiwan

**Keywords:** artificial intelligence, end-of-life care, machine learning, palliative care, survival prediction, terminal cancer, wearable device

## Abstract

**Background:**

An accurate prediction of mortality in end-of-life care is crucial but presents challenges. Existing prognostic tools demonstrate moderate performance in predicting survival across various time frames, primarily in in-hospital settings and single-time evaluations. However, these tools may fail to capture the individualized and diverse trajectories of patients. Limited evidence exists regarding the use of artificial intelligence (AI) and wearable devices, specifically among patients with cancer at the end of life.

**Objective:**

This study aimed to investigate the potential of using wearable devices and AI to predict death events among patients with cancer at the end of life. Our hypothesis was that continuous monitoring through smartwatches can offer valuable insights into the progression of patients at the end of life and enable the prediction of changes in their condition, which could ultimately enhance personalized care, particularly in outpatient or home care settings.

**Methods:**

This prospective study was conducted at the National Taiwan University Hospital. Patients diagnosed with cancer and receiving end-of-life care were invited to enroll in wards, outpatient clinics, and home-based care settings. Each participant was given a smartwatch to collect physiological data, including steps taken, heart rate, sleep time, and blood oxygen saturation. Clinical assessments were conducted weekly. The participants were followed until the end of life or up to 52 weeks. With these input features, we evaluated the prediction performance of several machine learning–based classifiers and a deep neural network in 7-day death events. We used area under the receiver operating characteristic curve (AUROC), *F*_1_-score, accuracy, and specificity as evaluation metrics. A Shapley additive explanations value analysis was performed to further explore the models with good performance.

**Results:**

From September 2021 to August 2022, overall, 1657 data points were collected from 40 patients with a median survival time of 34 days, with the detection of 28 death events. Among the proposed models, extreme gradient boost (XGBoost) yielded the best result, with an AUROC of 96%, *F*_1_-score of 78.5%, accuracy of 93%, and specificity of 97% on the testing set. The Shapley additive explanations value analysis identified the average heart rate as the most important feature. Other important features included steps taken, appetite, urination status, and clinical care phase.

**Conclusions:**

We demonstrated the successful prediction of patient deaths within the next 7 days using a combination of wearable devices and AI. Our findings highlight the potential of integrating AI and wearable technology into clinical end-of-life care, offering valuable insights and supporting clinical decision-making for personalized patient care. It is important to acknowledge that our study was conducted in a relatively small cohort; thus, further research is needed to validate our approach and assess its impact on clinical care.

**Trial Registration:**

ClinicalTrials.gov NCT05054907; https://classic.clinicaltrials.gov/ct2/show/NCT05054907

## Introduction

### Survival Prediction Tools in End-of-Life Care

Survival prediction is a critical aspect of end-of-life care. Knowing the likely clinical course allows the medical team to create care plans and avoid the overuse of aggressive care. It also helps patients and their families in other ways, such as by allowing the families to fulfill the patients’ final wishes. The most widely recognized and commonly used tools for survival prediction in end-of-life care include the Palliative Performance Scale [1-4], Palliative Prognostic Index [5-8], and Palliative Prognostic Score [9-12], which had been developed and validated in the past decades.

These tools typically rely on clinical symptoms, signs, and functional levels to estimate prognosis. Some tools incorporate blood tests and clinician predictions to enhance the evaluation process (see Multimedia Appendix 1 [1-16] for further detail). Although these tools have shown fair performance in predicting short-term survival lengths ranging from 7 to 60 days, studies validating these tools have generally been conducted in inpatient settings and have usually considered only a single evaluation upon the patient’s admission [17,18].

### The Need for Anticipating and Identifying the Dying Process in Outpatient Care

End-of-life care encompasses various scenarios and preferences. Many patients express a desire for home care, seeking a sense of safety and comfort during their final weeks of life [19-23]. However, this phase can involve a range of potential events, varying from mild discomfort to urgent medical needs to, ultimately, the death event.

The existing tools may perform well in predicting survival from a statistical and population perspective but fail to capture the diverse trajectories of individual patients. For example, patients with high prognostic scores may pass away more rapidly than predicted, whereas those with low scores may survive longer than expected. Given the emphasis on outpatient and home care to reduce hospitalization and alleviate the burden on patients and their families, it is crucial to anticipate and identify impending changes in advance, including the dying process. This allows for adequate preparation and support to be provided promptly.

### The Application of Wearable Devices and Artificial Intelligence in End-of-Life Care

Digital health technology has been widely adopted in end-of-life care for various purposes, such as education, telemedicine, and prognosis prediction using electronic health records [24-31]. Although wearable devices have also been demonstrated to help monitor and predict physical conditions [32-40], there have been only a few studies on the use of wearable devices in end-of-life care. One study focused on the feasibility and acceptability of the devices [41,42], whereas another reported that a deep learning model could predict future outcomes using noncommercial actigraphy data in an in-hospital setting [43]. We believe that the prediction of death or emergent events using wearable devices and artificial intelligence (AI) could enable the medical team to provide timely and high-quality care. However, there is currently no research examining the use of commercial wearable devices in a general setting.

This pilot study aimed to combine wearable devices and machine learning to develop a prediction model for the death event of patients with cancer at the end of life. We hypothesized that using a smartwatch for continuous monitoring may provide greater insight into the progress of patients with terminal cancer and, with the aid of machine learning techniques, may be able to predict changes in their conditions.

## Methods

### Study Design and Participants

This is a prospective observational pilot study conducted at the National Taiwan University Hospital from September 2021 to August 2022. Patients who were receiving or going to receive outpatient or home-based end-of-life care were referred by their medical staff. The eligibility criteria were (1) age >20 years and (2) terminal cancer diagnosis (incurable cancer with limited life expectancy, judged by the physician of the primary team). The exclusion criterion was the inability to use smartphones because patients or their caregivers needed to use a smartphone to upload the wearable device data.

### Ethics Approval

The study protocol was approved by the institutional review board of the National Taiwan University Hospital (RIND202105097) and registered on ClinicalTrials.gov (NCT05054907).

### Data Collection

The following data were collected during the study: basic demographic data, clinical assessment data, and wearable device data. Basic demographic data were evaluated upon enrollment, including age, sex, cancer diagnosis, presence of metastasis, systemic disease, and material use.

Clinical assessments were conducted on a weekly basis, evaluating consciousness, appetite, symptoms, functional level (using the Australia-modified Karnofsky Performance Status [AKPS]) [44], and clinical care phase [45] (Multimedia Appendix 2 [44,45]). The evaluation was usually performed by the research assistant face to face or via a phone call, depending on whether the patient had a clinic visit that week. However, the patient’s condition was assessed by the inpatient care staff if they were admitted to the hospital or by the home care team if they received a home visit that week.

All participants were provided with a smartwatch, Garmin VivoSmart 4 (Garmin), and asked to wear it all day as long as they could tolerate it. The wearable device data were collected continuously. Participants or their caregivers were taught to operate a smartphone app to synchronize the wearable data on a regular basis (at least once every 7 days). Physiological data, including steps walked, heart rate (HR), sleep status, and blood oxygen saturation (measured during sleep time), were collected. The data were presented as a total sum or an average value of 1 day.

Most patients who received end-of-life care in Taiwan had a life expectancy of just a few weeks or months [46]. To ensure that we could follow most of these patients until the end of their lives, we set a follow-up duration of up to 52 weeks. We hypothesized that physiological changes, which can be detected through wearable device measurements, may occur within a shorter time frame, ideally less than 2 weeks before the death event [47]. Consequently, we chose a 7-day prediction interval, as it is an intuitively manageable time frame that enables clinicians and caregivers to communicate essential information to families in advance.

### Data Processing

**Figure 1:**
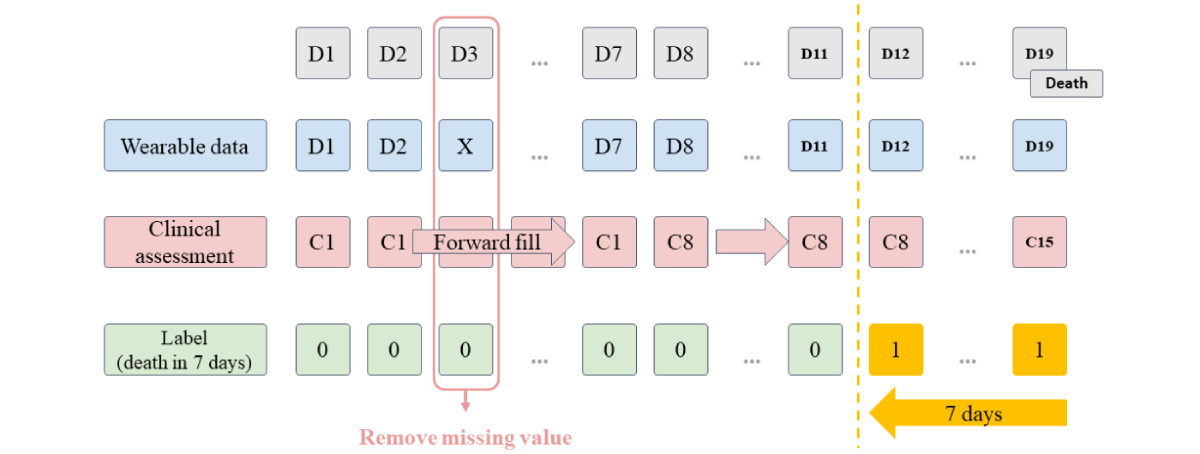
The combination of wearable device data and clinical assessments. The figure illustrates the process of data combination. Each column represents 1 data point. Each row represents a different kind of data. Days without any wearable device data uploaded were directly excluded from the analysis.

The data set was a combination of basic demographic data, clinical assessment data, and wearable device data. As shown in Figure 1, one day corresponded to one data point, which served as an individual observation for model training. The label was a recent death event, defined based on whether the patient died within the next 7 days. As the clinical assessment was performed once a week, we used forward filling until the next assessment. For any data point of any case, if some of the wearable data were missing, we used interpolation to fill in the missing value. However, days without any wearable device data uploaded were directly excluded from the analysis.

**Figure 2:**
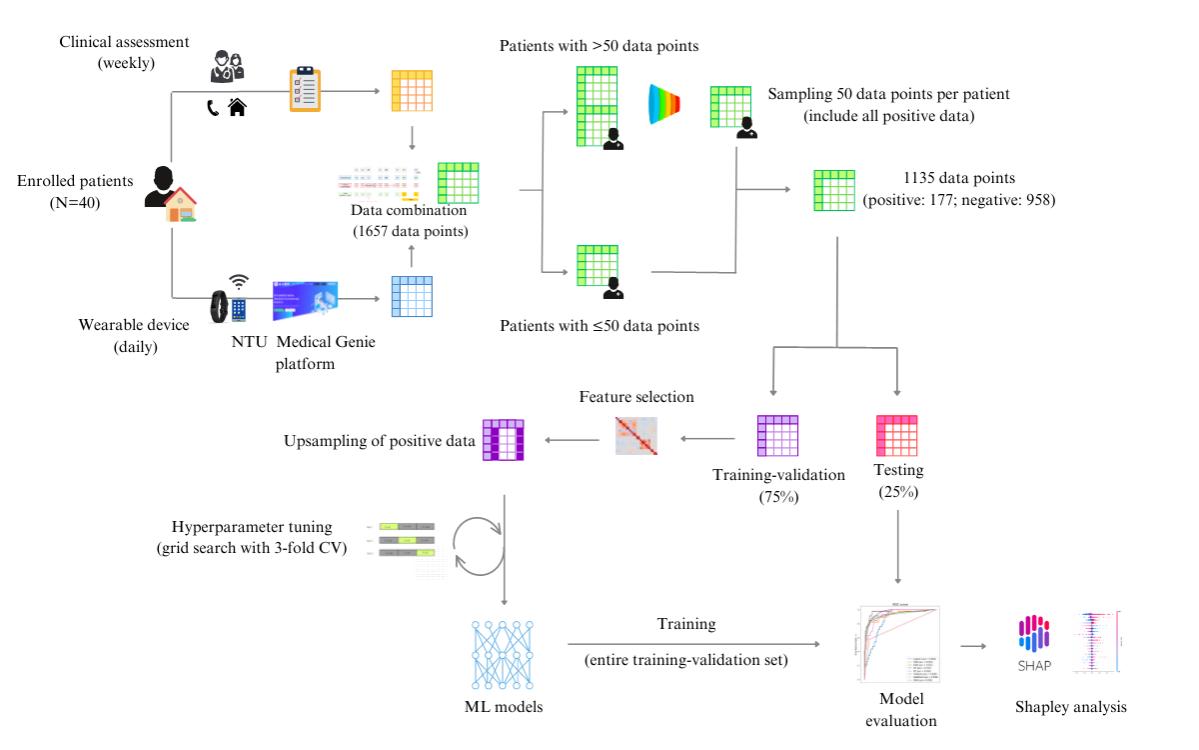
Flowchart of the study. The figure illustrates the flowchart of the entire study from data collection and data processing to the training and evaluation of machine learning models. CV: cross-validation; ML: machine learning; NTU: National Taiwan University.

For cases that received a follow-up period longer than 50 days, we kept all the data labeled as positive and randomly sampled the remaining negative data points to bring the total number of data points to 50 in each case. For cases with a follow-up period shorter than 50 days, we included all of the data points. The data set was then divided into testing (25%) and training-validation (75%) sets, stratified by label and sex. To improve the model training, we upsampled the positive data points in the training-validation set. The entire flowchart of the study is shown in Figure 2.

### Feature Engineering

The original features collected are listed in Textbox 1. To account for changes in an individual’s condition, we calculated the differences between the original features and their past averages in wearable data and clinical data and used these differences as new features.

For the feature selection, 2 demographic features (sex and age) were directly included to account for the physical differences between the individuals. Most wearable device features were directly included in the model, except for 3. “Sleep duration” parameters were transformed into the ratio of wakefulness to sleep. “Stress” parameters were excluded owing to there being no clear definition of their value. “Resting heart rate” data were excluded considering the similarity of “resting heart rate” to “minimal heart rate” and a lack of a clear definition of “resting.” As for clinical assessment features, we used the *SelectKBest* method built into the *Scikit-learn* package and selected the top 10 potential candidate features using ANOVA on the training set. A correlation analysis between the selected features was performed on the training-validation set.

**All the original features collected in the study.**

**Physiological factor**

- Steps
- Minimum heart rate (HR)
- Maximum HR
- Average HR
- Resting HR
- Average stress level
- Maximum stress level
- Sleep duration
- Deep sleep duration
- Light sleep duration
- Rapid eye movement (REM) sleep duration
- Awake duration
- SpO_2_ (expressed in average)

**Clinical assessment**

- Consciousness
- Appetite
- Urination
- Edema
- Pain score
- Sleep
- Drowsiness
- Nausea
- Constipation
- Diarrhea
- Dyspnea
- Fatigue
- Fever
- Functional level (using Australia-modified Karnofsky Performance Status)
- Care phase
- Pain control change

**Basic demographic**

- Sex
- Age
- Cancer diagnosis
- Diagnosis time
- Confirmed metastasis
- Past history
- Alcohol use
- Betel nut use
- Cigarette use

### Classification Model

We chose the following machine learning–based classifiers for supervised learning: logistic regressions, support vector machine, decision trees, random forests, k-nearest neighbor (KNN), adaptive boosting (AdaBoost), and extreme gradient boosting (XGBoost). We proposed a multiperceptron deep neural network to compare the performance of deep learning models with that of machine learning models on death prediction. The models were implemented using the Python library *Scikit-learn*.

### Model Assessment

Three-fold cross-validation was used in the training-validation set for hyperparameter tuning. The grid search parameters are listed in Table 1. Once the optimal hyperparameters were selected, we trained each model on the full training-validation set and evaluated its performance on the testing set. We used the area under the receiver operating characteristic curve (AUROC), precision, recall (sensitivity), *F*_1_-score, specificity, and accuracy as evaluation metrics. To reduce the impact of randomness on the algorithms, we repeated the training process 100 times for each model and reported the average values of the evaluation metrics. To investigate the impact of clinical assessment on prediction, we also trained and evaluated the models using only wearable device parameters, sex, and age as input features.

**Table 1:** Grid search parameters in hyperparameter tuning.

Classifier^a^	Parameters_grid	Selected value
LogisticRegression	Penalty: [“l1,” “l2,” “elasticnet,” “none”] C: {1, 0.1, 0.01} Solver: [“lbfgs,” “newton-cg,” “liblinear,” “sag,” “saga”]	Penalty=“11” C=0.1 Solver=“liblinear”
SVM^b^ (kernel = “rbf,” degree = 3)	C: [0.1, 1, 10, 100] Gamma: [1, 0.1, 0.01, 0.001]	C=1 Gamma=1
DecisionTree	criterion: [“gini,” “entropy”] max_depth: [None, 4, 5, 6, 7, 8] min_samples_split: {2, 4, 8, 10, 20, 30, 40}	Criterion=“entropy” Max_depth=None Min_samples_split=2
RandomForest	n_estimators: {30, 100, 200} max_depth: {4, 5, 6, 7, 8} min_samples_split: {2, 4, 6, 8} min_samples_leaf: {1, 2, 3}	n_estimators=100 Max_depth=8 Min_samples_split=2 Min_samples_leaf=1
KNeighborsClassifier	n_neighbors: {5, 10, 15, 20, 30} weights: [“uniform,” “distance”] metric: [“minkowski,” “euclidean,” “manhattan”]	n_neighbors=5 weight=“distance” metric=“manhattan”
AdaBoostClassifier	n_estimators: {5, 10, 30, 50, 100, 500} learning_rate: [0.01, 0.05, 0.1, 0.15, 0.2, 0.3, 0.5, 1.0] algorithm: [“SAMME,” “SAMME.R”]	n_estimator=500 learning_rate=1.0 algorithm=“SAMME.R”
XGBClassifier (eval_metric = “aucpr,” n_estimators = 100, booster = “gbtree,” colsample_bytree = 1, learning_rate = 0.3)	eta: [0.01, 0.05, 0.1, 0.15, 0.2, 0.3] gamma: {0, 1, 5} max_depth: {5, 6, 8, 10} min_child_weight: {0, 1, 2, 5, 10}	min_child_weight=0 max_depth=5 gamma=0 eta=0.2
MLPClassifier (hidden_layer_sizes = (64, 64, 64), activation = “relu”)	solver: [“adam,” “lbfgs”] alpha: [0.0001, 0.001, 0.01, 0.05, 0.1] learning_rate: [“constant,” “adaptive”] learning_rate_init: [0.01, 0.005, 0.001] batch_size: {150, 300, 500}	Solver=“lbfgs” Alpha=0.1 Learning_rate=“constant” Batch_size=150 Learning_rate_init=0.01

^a^The algorithms were performed using the Python package *Scikit-learn* 0.24.2, and all other parameters not shown in this table were set to their default values.

^b^SVM: support vector machine.

### Model Explanation

To explore the machine learning model more deeply, a Shapley additive explanations (SHAP) algorithm was applied [48,49]. The impact of the feature values on each prediction of a recent death event in the testing set was obtained.

### User Feedback on Wearable Devices

To evaluate the feasibility of wearable devices in the population with terminal cancer, we surveyed the participants during weekly follow-ups to assess any difficulties or discomfort they experienced while using the wearable device. At the end of the study, we also solicited feedback from the family or main caregivers of the participants who had passed away about their experience with the wearable device and their willingness to use similar devices in the future.

## Results

### Patient Demographics

Between September 2021 and August 2022, a total of 45 patients were enrolled. Data from 11% (5/45) of patients were unavailable; therefore, these patients were excluded from the data analysis: 4% (2/45) left the study early owing to personal reasons, 4% (2/45) died soon after the enrollment, and 2% (1/45) did not use the wearable device owing to a personal reason. In all, 89% (40/45) of patients were included in the analysis.

Table 2 illustrates the demographics of the study participants, with approximately one-third (12/45, 27%) being diagnosed with lung cancer. The median age of the patients was 70.5 years. At the time of enrollment, most participants had limited function, as determined by the AKPS assessment. The median care duration was 34 days, ranging from a minimum of 6 days to a maximum of 271 days. Except for 5% (2/45) of patients who withdrew from the study for personal reasons, most patients were followed until their death, at which point their care time was equal to their survival period.

**Table 2:** Basic demographics of the participants (n=40).

Characteristic		Participant, n (%)
**Age group** **(years)**		
	≤40	1 (2)
	41-65	12 (30)
	65-80	15 (38)
	≥80	12 (30)
**Sex**		
	Male	17 (42)
	Female	23 (58)
**Cancer diagnosis**		
	Lung	12 (30)
	Colorectal	6 (15)
	Head and neck	5 (12)
	Pancreas	4 (10)
	Liver	3 (8)
	Breast	2 (5)
	Prostate	2 (5)
	Other	6 (15)
**AKPS^a^ score on initial assessment **		
	≤20	12 (30)
	30-50	20 (50)
	60-70	7 (18)
	≥80	1 (2)
**Follow-up time or survival length^b^**		
	<7 days	2 (5)
	8-30 days	14 (35)
	1-2 months	15 (38)
	2-3 months	5 (12)
	>3 months	4 (10)
**Wearable device use (%)**		
	<25	3 (8)
	25-50	3 (8)
	50-75	6 (15)
	75-90	9 (22)
	>90	19 (48)
Mortality during follow-up time^b^		38 (95)
Participants whose wearable device data were collected until at least 7 days before their death events		28 (70)

^a^AKPS: Australia-modified Karnofsky Performance Status.

^b^Overall, 2 (5%) patients left the study for personal reasons. Most patients were followed until their death, for whom the follow-up time represented their survival period.

### Completeness of Data

The wearable devices were worn, on average, for 77.42% (n=1657) of the total 2140 study days. Of the 40 participants, 34 (85%) wore the devices for more than half of their study days. Moreover, 28 (70%) out of 40 participants wore the devices until at least 7 days before their deaths. Sleeping-related parameters were missing for approximately 14.67% (243/1657) of the collected data points, which indicates that the devices were not worn at night on those days.

Figure 2 shows the study flowchart and data processing. A total of 1657 data points were collected, of which 177 (10.68%) were labeled as positive. After a downsampling of the participants with follow-up time longer than 50 days, a total of 1135 (68.5%) out of 1657 data points were included, with 177 (15.59%) positive labels. One-fourth of the data (284/1135, 25.02% data points) were randomly assigned to the testing set, and the remaining were assigned to the training set. Random upsampling of the positive data points resulted in a total of 1436 data points in the training-validation set, with an equal number of positive and negative data points.

### 7-Day Death Event Prediction Model

**Figure 3:**
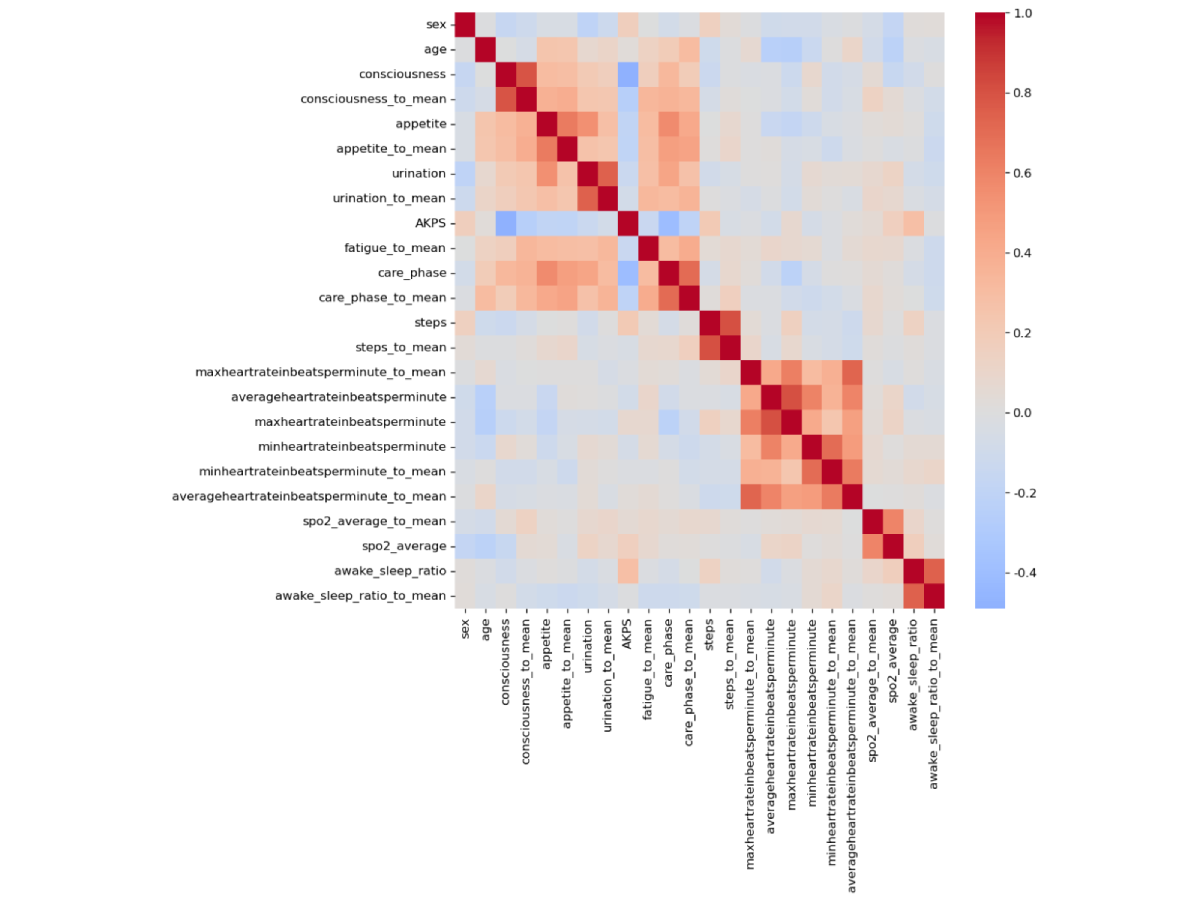
The correlation matrix of the selected features on the training-validation set. Features with name ending with “_to_mean” represent the calculated difference between the original feature and past 4 week average in clinical assessment and between the original feature and past 7 day average in wearable device data. AKPS: Australia-modified Karnofsky Performance Status.

A total of 24 features were included in the model (Table 3). The correlation matrix of all the selected features in the training-validation set is shown in Figure 3.

**Figure 4:**
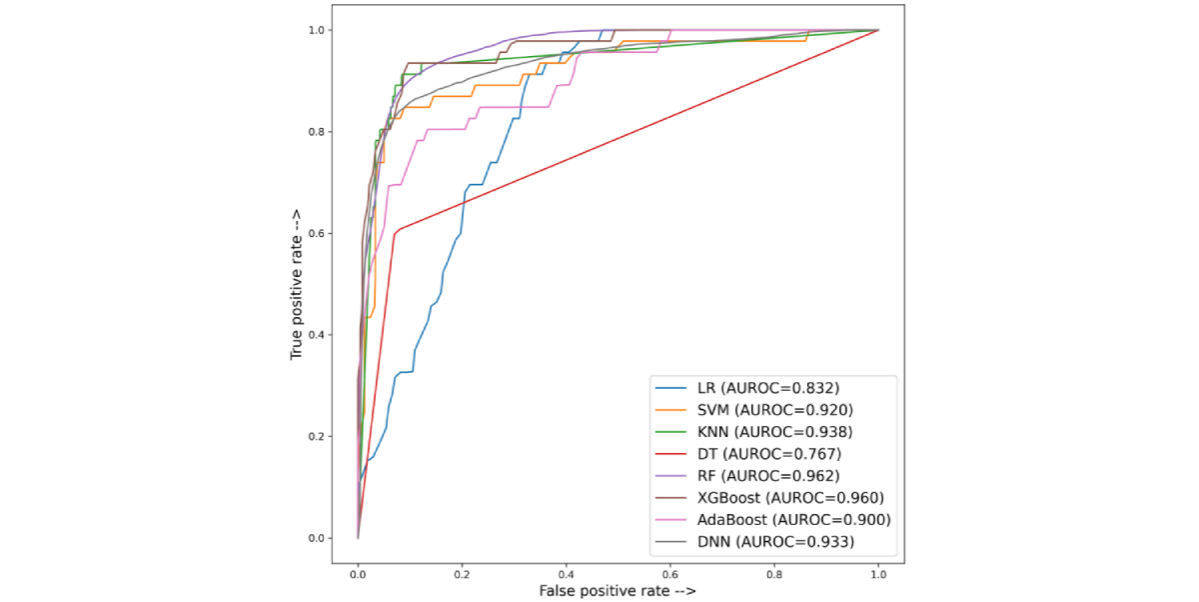
Area under the receiver operating characteristic curve (AUROC) of each model on the testing set. AdaBoost: adaptive boosting; DNN: deep neural network; DT: decision tree; KNN: k-nearest neighbor; LR: logistic regression; RF: random forest; SVM: support vector machine; XGBoost: extreme gradient boost.

Figure 4 and Table 4 show the performance of the machine learning models in terms of the AUROC and other evaluation metrics. Among all the models, XGBoost yielded the best result, with an AUROC of 96%, an *F*_1_-score of 78.5%, an accuracy of 93%, and a specificity of 97% on the testing set, whereas deep neural network achieved an AUROC of 93.3%, an *F*_1_-score of 76.8%, an accuracy of 92.7%, and a specificity of 96.2%.Random forests and KNN also yielded fair performances. The result of the models without any clinical assessment features is shown in Table 5.

**Table 3:** Selected features in the models.

	Feature^a^
Basic demographic	“sex” “age”
Clinical assessments	“consciousness” “consciousness_to_mean” “appetite” “appetite_to_mean” “urination” “urination_to_mean” “AKPS” “fatigue_to_mean” “care_phase” “care_phase_to_mean”
Wearable device parameters	“steps” “steps_to_mean” “maxheartrateinbeatsperminute” “maxheartrateinbeatsperminute_to_mean” “averageheartrateinbeatsperminute” “averageheartrateinbeatsperminute_to_mean” “minheartrateinbeatsperminute” “minheartrateinbeatsperminute_to_mean” “spo2_average”^b^ “spo2_average_to_mean” “awake_sleep_ratio” “awake_sleep_ratio_to_mean”

^a^Features with name ending with “_to_mean” represent the calculated difference between the original feature and past-4-week average in clinical assessment and between the original feature and past-7-day average in wearable device data.

^b^“Spo2_average” represents the average blood oxygen saturation measured during the nighttime.

**Table 4:** Model performances (based on evaluation metrics) on the testing set.

Model name	Accuracy	Precision	Recall	*F*_1_-score	Specificity	AUROC^a^
LR^b^	0.750	0.360	0.720	0.540	0.750	0.832
SVM^c^	0.860	1.000	0.130	0.565	1.000	0.920
KNN^d^	0.910	0.660	0.910	0.785	0.910	0.938
Decision tree	0.877	0.624	0.605	0.615	0.930	0.767
Random forest	0.923	0.777	0.733	0.755	0.959	0.962
XGBoost^e^	0.930	0.850	0.720	0.785	0.970	0.960
AdaBoost^f^	0.900	0.700	0.650	0.675	0.950	0.900
DNN^g^	0.927	0.790	0.746	0.768	0.962	0.933

^a^AUROC: area under the receiver operating characteristic curve.

^b^LR: logistic regression.

^c^SVM: support vector machine.

^d^KNN: k-nearest neighbor.

^e^XGBoost: extreme gradient boost.

^f^AdaBoost: adaptive boosting.

^g^DNN: deep neural network.

**Table 5:** Model performance without the clinical assessment features.

Model name	Accuracy	Precision	Recall	*F*_1_-score	Specificity	AUROC^a^
LR^b^	0.720	0.350	0.870	0.610	0.690	0.814
SVM^c^	0.840	0.600	0.070	0.335	0.990	0.824
KNN^d^	0.800	0.430	0.700	0.565	0.820	0.821
Decision tree	0.835	0.494	0.530	0.512	0.894	0.712
Random forest	0.868	0.610	0.501	0.556	0.938	0.893
XGBoost^e^	0.890	0.720	0.500	0.610	0.960	0.868
AdaBoost^f^	0.830	0.470	0.570	0.520	0.880	0.815
DNN^g^	0.893	0.687	0.632	0.660	0.944	0.899

^a^AUROC: area under the receiver operating characteristic curve.

^b^LR: logistic regression.

^c^SVM: support vector machine.

^d^KNN: k-nearest neighbor.

^e^XGBoost: extreme gradient boost.

^f^AdaBoost: adaptive boosting.

^g^DNN: deep neural network.

### Explainable AI

**Figure 5:**
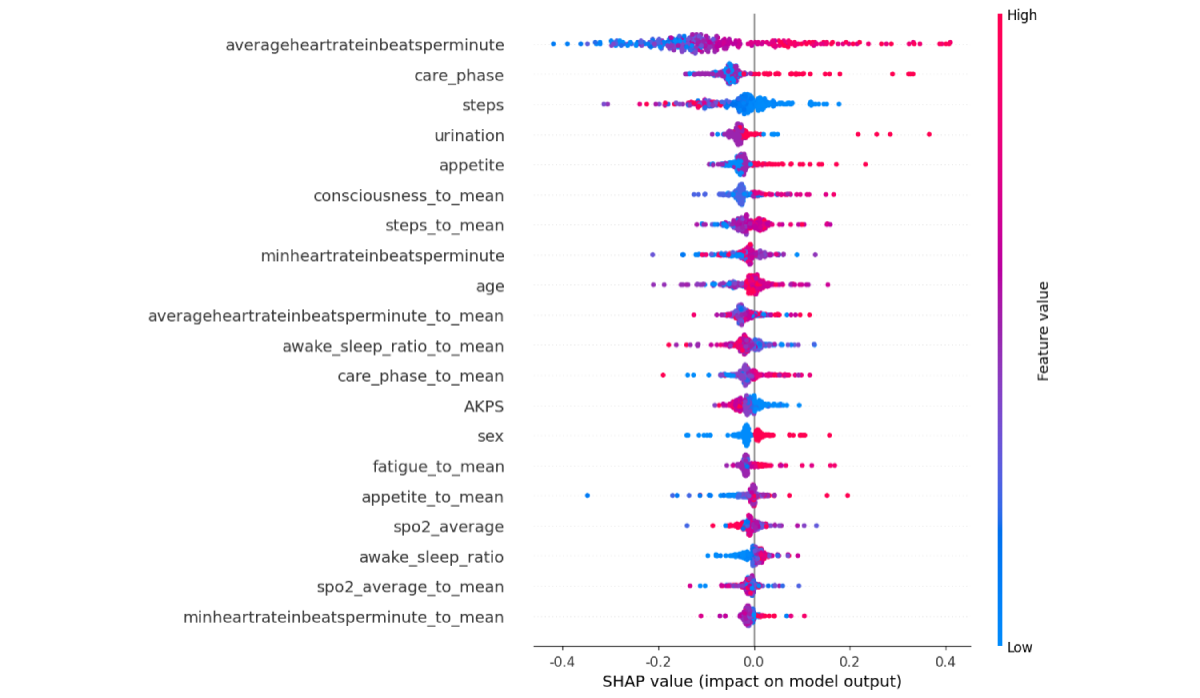
The Shapley additive explanations (SHAP) value of the extreme gradient boosting model on the testing set. The summary plot illustrates the feature values and the SHAP values of individual points. Features with name ending in “_to_mean” represent the calculated difference between the original feature and past 4 week average in clinical assessment and between the original feature and past 7 day average in wearable device data. AKPS: Australia-modified Karnofsky Performance Status.

Figure 5 and Table 6 show the results of the SHAP value analysis for the top-performing model, XGBoost, on the testing set. We also analyzed the SHAP values for the random forest, KNN, and deep learning models, which demonstrated similar performance levels (the results are shown in Multimedia Appendix 3). Among these models, the feature “average heart rate” consistently had the highest impact on prediction. Other features that were ranked in the top 5 by at least 2 of the models included “care_phase,” “urination,” “appetite,” “sex,” and “steps.”

**Figure 6:**
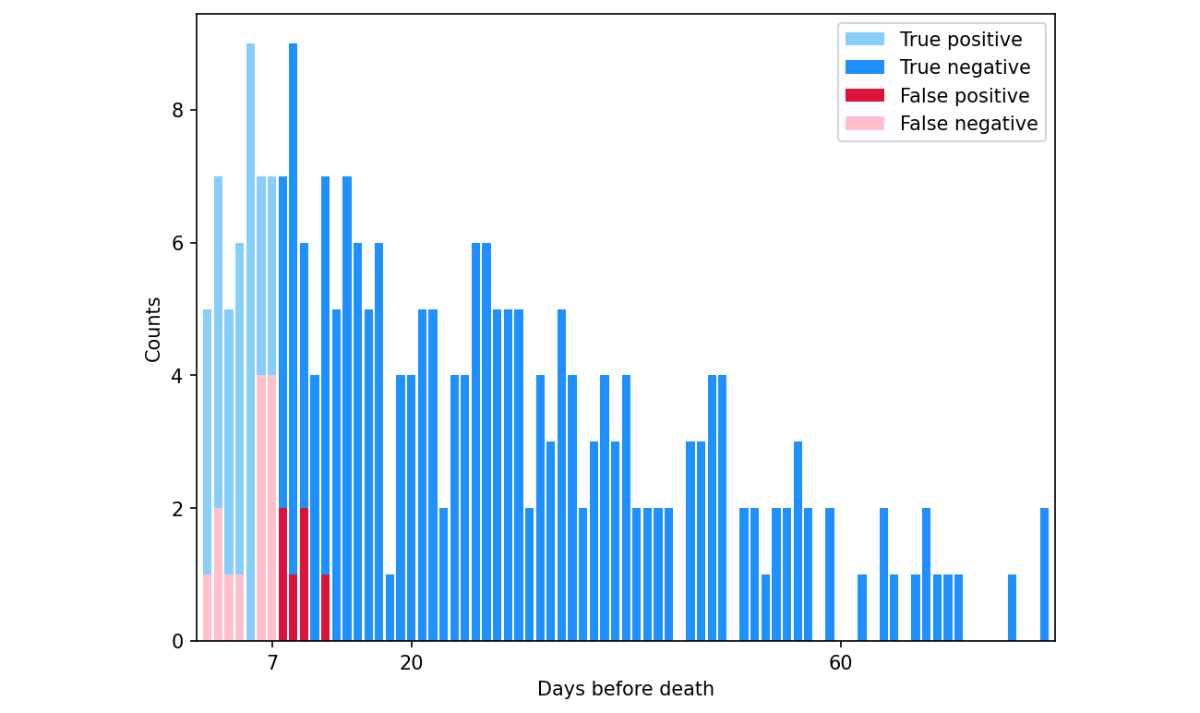
Testing data distribution based on time before death (extreme gradient boosting). This figure illustrates a bar plot depicting the distribution of data based on the time before death. The x-axis represents the time before death events in days, whereas the y-axis represents the count of specific prediction results. Within the 7-day range, the data points were labeled as positive, resulting in either true-positive or false-negative outcomes. Conversely, data points occurring >8 days before death were labeled as either true-negative or false-positive outcomes. Therefore, the pink and red bars in the figure represent the distributions of false-negative and false-positive cases, respectively, as predicted by extreme gradient boosting.

**Figure 7:**
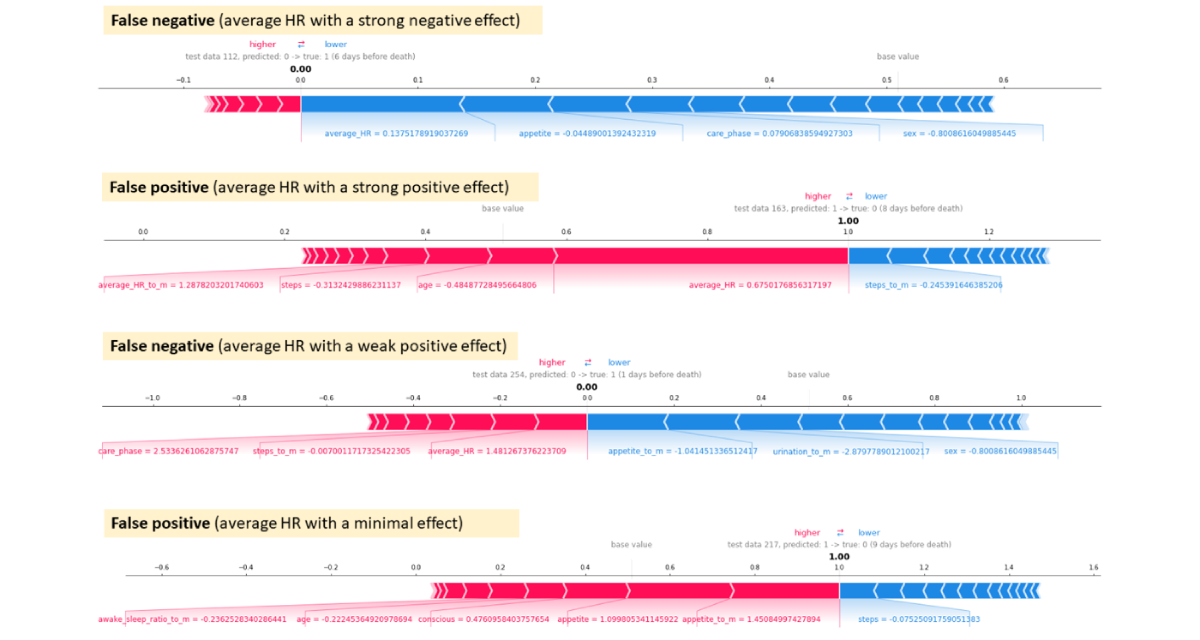
The Shapley additive explanations (SHAP) value analysis of 4 wrong prediction cases. This figure shows the SHAP value analysis of 4 wrong prediction cases. The upper 2 cases demonstrate the substantial impact of the average heart rate on the prediction, resulting in false-negative or false-positive outcomes. By contrast, the lower 2 cases exhibit the minimal or negligible effects of the average heart rate, with other factors dominating the prediction results. Note: features with name ending in “_to_m” represent the calculated difference between the original feature and past 4 week average in clinical assessment and between the original feature and past 7 day average in wearable device data. HR: heart rate.

For the best performing model, XGBoost, the distribution of the testing set data points in terms of “time before death events” and the prediction results is shown in Figure 6. A few samples of the SHAP analysis in the wrong prediction cases are shown in Figure 7.

**Table 6:** The mean absolute Shapley additive explanations (SHAP) value of the extreme gradient boosting model on the testing set.

Feature	Mean absolute SHAP value (SD)
averageheartrateinbeatsperminute	0.1454 (0.088)
care_phase	0.0600 (0.040)
steps	0.0533 (0.054)
urination	0.0356 (0.032)
appetite	0.0328 (0.025)
consciousness_to_mean	0.0307 (0.023)
steps_to_mean	0.0273 (0.025)
minheartrateinbeatsperminute	0.0269 (0.028)
age	0.0268 (0.034)
averageheartrateinbeatsperminute_to_mean	0.0265 (0.017)
awake_sleep_ratio_to_mean	0.0248 (0.026)
care_phase_to_mean	0.0231 (0.023)
AKPS^a^	0.0218 (0.016)
sex	0.0217 (0.023)
fatigue_to_mean	0.0193 (0.017)
appetite_to_mean	0.0175 (0.033)
spo2_average	0.0163 (0.018)
awake_sleep_ratio	0.0157 (0.016)
spo2_average_to_mean	0.0143 (0.017)
minheartrateinbeatsperminute_to_mean	0.0140 (0.013)
urination_to_mean	0.0134 (0.021)
consciousness	0.0119 (0.012)
maxheartrateinbeatsperminute	0.0077 (0.010)
maxheartrateinbeatsperminute_to_mean	0.0077 (0.008)

^a^AKPS: Australia-modified Karnofsky Performance Status.

### User Feedback on Wearable Devices

User feedback was collected when the patient left the study, mostly owing to death. All the participants relied on their caregivers to update the data from the wearable device and charge the device. The demographics of the caregivers and feedback details are presented in Table 7 and Textbox 2. Overall, 30% (12/40) of the caregivers were aged ≥65 years, and only 32% (13/40) had experience in using wearable devices. Most of the caregivers could operate the device well after being instructed, and 70% (28/40) of the participants expressed willingness to participate in a similar study in the future. The most frequently reported problem was forgetting to charge the wearable device, which needed to be done once every 5 days in our study. Redness and itchiness of the skin were the main side effects reported, but only in a few patients (2/40, 5%).

**Table 7:** Demographics of the caregivers (n=40) and feedback on wearable device use.

Characteristic		Participants, n (%)
**Age group (years)**		
	<35	3 (8)
	35-50	8 (20)
	50-65	17 (42)
	65-80	10 (25)
	>80	2 (5)
**Educational level**		
	Elementary school	6 (15)
	Junior high school	4 (10)
	Senior high school	9 (22)
	College or university	20 (50)
	Unclear	1 (2)
**Previous experience in using wearable devices**		
	Yes	13 (32)
	No	27 (68)
**Cooperation with wearable device and smartphone use**		
	No problem with manipulating the device or apps	30 (75)
	Need some help but generally fine	8 (20)
	Need a lot of help or completely unable to cooperate	2 (5)
**Willing to participate in a similar study in the future (answer from the caregiver or wearable device user)**		
	Yes	28 (70)
	No	12 (30)
**Problem when using the device**		
	Forget to charge the device	6 (15)
	Discomfort (itch or redness) on the skin	2 (5)
	Forget to wear	2 (5)

**Opinions and feedback regarding the wearable device and the study.**

- “Want a larger monitor on the watch.”
- “Want to directly manipulate and see data on the watch.”
- “Hope more detailed description on the parameter, and thus to help the user understand its meaning.”
- “Patient felt bothered by the vibration of watch.”
- “Not wearing the device since the patient is in delirium and kept scratching the wristband unconsciously.”
- “Not wearing due to admission and wrist was inserted with IV catheter.”
- “Not wearing due to admission and there is only foreign caregiver at bedside. Families could not help charge the watch or update the data.”

## Discussion

### Principal Findings

Although we are not the first to explore the use of wearable devices in end-of-life care, we are unique in our approach. Pavic et al [41,42] demonstrated the feasibility of wearable devices and compared the data of patients with readmission with those of patients without readmission. Yang et al [43] used actigraphy along with deep learning to predict the prognosis of patients who were hospitalized. However, actigraphy recorded only wrist movements, and their study was confined to a hospital setting. Our study extends this area of research by developing prediction models using data from commercially available wearable devices in a broader, general end-of-life care setting. In addition, we demonstrated the potential of AI in predicting death events among patients with cancer at the end of life.

### Wearable Device Parameters in Death Event Prediction

Our study has proved that the physiological data measured by a wearable device can be used in clinical prediction models. The SHAP values further provide explanations for the predictions of the models. Among all the parameters in our study, “average heart rate” was identified as the most important feature in predicting a recent death event. This is consistent with previous studies, as an increased HR was noticed before an emergency visit to a hospital or in the last days of patients with a terminal illness [41,47].

Besides HR, Pavic et al [41] also found that “heart rate variation” and “steps speed,” but not “steps count,” were significantly different between patients with and patients without emergency visits to hospitals. Contrary to their result, “steps count” was identified as an indicating parameter in some of our models. We suspect that the difference came from the relatively poor functional level of our population. Ideally speaking, individuals who are ambulatory would be the most suitable participants for wearable device studies, as certain parameters such as “steps count,” “steps speed,” and “climbing stairs” could yield more diverse and enriching data in these populations. However, to faithfully reflect the demographics that we typically care for, we did not establish any exclusion criteria based on the functional level. As a result, most participants in our study were patients who were not ambulatory, as evidenced by their AKPS scores upon enrollment—over 80% (32/40) of the patients scored <50.

Although our findings may have limitations when applied to individuals with better functional status, we believe that the results are pertinent and applicable to the patients we typically care for. Interestingly, even though our patients had limited functional abilities, we discovered that the ambulatory parameter “steps” still held predictive value for imminent mortality. This could indirectly reflect the patient’s status in very basic activities, such as changing posture, going to the bathroom, and sitting up. Patients tend to stay in bed more and decrease these activities when they enter the terminal phase.

The wearable device used in our study can measure blood oxygen saturation (SpO_2_), which is an increasingly common function for new wearable devices in the market. As it has been reported that saturation decreases in the last days of patients [47], it is a little surprising that the parameter does not seem to have a role in any of our models. A reasonable explanation for this is varied data quality. Until now, there have been no commercial smartwatches or wristbands that are well validated for measuring SpO_2_ [50]. Aside from improving the quality of hardware measurement, a possible solution is to consider the change and variation in oxygen saturation on a second or minute scale, rather than the daily average value. Deep learning models for high-dimensional time-series data, such as convolutional neural network and long short-term memory, provide good performances for this type of data [43,51,52] and will be considered in our future work.

### Clinical Assessments in Death Event Prediction

As our explainable AI models have shown, clinical assessment features play a role in predicting death events. Among the models with a good performance, we found that a sign of reduced urine output, poor appetite, and deteriorating clinical care phase suspected by the clinical caregiver were ranked as high-impact features.

To further investigate the impacts, we reran the models using only wearable device parameters, sex, and age as input features. This resulted in lower precision and recall in predicting events while maintaining fair specificity, as shown in Table 5. When building the data set, we used forward filling in clinical assessment data to ensure that it reflects the most recent evaluation of the patient’s condition at that time point. In our opinion, the clinical assessment features might help the models determine whether the physiological changes detected by the wearable device are meaningful indicators of a potential event. These results suggest that clinical assessment is just as important as wearable device monitoring.

### Wrong Prediction Case Analysis

We further examined the worst case of the XGBoost model prediction. First, we examined the distribution of “time before death events” in our testing set and identified that all the wrong predictions fell within 2 ranges of time: 1 to 7 days (false negatives) and 7 to 12 days (false positives) before death, as shown in Figure 6. These results reinforce the high specificity of our model, as patients who were far from the death event (eg, 20 days or longer before death) were rarely misclassified as “going to die.” Among the 284 data points in the testing set, only 6 (2.1%) false-positive predictions occurred, and in all cases, the patients died within 12 days.

During the review of the SHAP value analysis for incorrect predictions, 2 main types of explanations emerged, with the key feature being the “average heart rate.” Among the 6 false-positive predictions, 3 (50%) were primarily influenced by an elevated average HR, whereas the other 3 (50%) were driven by factors such as a decreased appetite or deteriorating care phase. A similar pattern was observed for the false-negative predictions within 1 to 7 days before death. In approximately half of the cases, a relatively low average HR played a significant role in predicting a negative outcome, accompanied by other parameters, including “care phase,” “appetite,” “consciousness,” and “steps,” aligning in the same direction. In the remaining half of the cases, the average HR had a small positive impact, whereas the other parameters contributed to a negative prediction. Some case results are depicted in Figure 7, and a comprehensive analysis of all the incorrect cases can be found in Multimedia Appendix 4.

Our analysis suggests that the average HR is an important indicator but not an absolute one in the 7-day death prediction model. In a vital sign study conducted in a palliative care unit, an increasing trend in HR was observed up to 2 weeks before death [47]. An increased HR may indicate a natural dying process, but it could also signify complications such as occult infection or sepsis. However, this trend was not observed in all patients. Conversely, certain causes of death, such as cardiac death, may be very acute events and not exhibit signs days before death. Different causes of death may contribute to the model’s false predictions.

### Unknown Interactions Between Parameters and Models

It is interesting to investigate whether age plays a role in the prediction. We observed that the average HR had more positive impacts on the prediction results in the younger patient group (aged <65 years). When reviewing the wrong predictions made for the data points belonging to younger patients, especially for the false-negative cases, we found that the average HR increased in all cases, leading to a small positive impact on the prediction of death events. As for the false-negative cases from patients aged >80 years, only 1 (25%) out of the 4 cases showed a small positive impact from the average HR, whereas the other 3 (75%) cases had a strong negative impact from it. This finding aligns with the well-known concept that the maximal HR and HR stress response decrease with age [53,54]. However, the model still ranked the average HR as the most important feature in both the aged 65-80 years and aged >80 years patient groups. It remains unclear whether there are more relationships between age and other parameters. Our analysis regarding different age groups is shown in Multimedia Appendix 5.

Although explainable models can provide high-impact features and, therefore, seem to be more reliable for clinical use, there have been debates over the use of explainable AI in health care [55]. One issue with these models is that they may not always provide accurate or clinically reasonable interpretations of data. For instance, in our deep learning model, the feature “sex” was ranked as having a high impact, and male data points had a higher SHAP value. However, this does not necessarily mean that male patients are more likely to die within 7 days, as our data were stratified by sex. There may be interactions between the “sex” feature and other features that contribute to the model’s prediction, but we lack the means to adequately explore this. Current explanation methods are approximations of a model’s decision-making process and may not accurately reflect the true underlying logic. Therefore, it is important to interpret the SHAP values and other explanations from explainable AI models with caution.

### Feasibility of Wearable Devices and AI in End-of-Life Care

Based on the feedback from our users, caregivers play a crucial role in the operation of these devices, as many of our patients were too weak to operate the devices as their illness progressed. However, to our surprise, many of them had no difficulty using the devices and expressed willingness to participate in similar studies in the future. Despite this, the results showed that, in the 38 patients who passed away during their follow-up time, only 28 (74%) out of wore their devices until 7 days before their death. This suggests that there are still many situations in which wearable devices become a burden for patients at the end of their lives. For example, patients may continuously try to remove the devices when they are in a delirium state. Another scenario is that when patients need to be admitted to the hospital, their family caregivers may not be able to stay with them and help charge the device or update the data. In 1 instance, although uncommon in end-of-life care, a caregiver reported having to remove the device because the patient had intravenous catheters on both hands while in the hospital. These issues highlight the limitations of the device hardware for these patients, but we expect these limitations to decrease as wearable technology continues to advance in size, form, and function.

### Potentials of Environmental Factors in Predicting the End-of-Life Status

In addition to the clinical conditions and personal physiological changes detectable through wearable device data, environmental factors have been shown to benefit the models in predicting the disease status [32]. It is intriguing to explore whether external environmental variations, such as temperature or air quality, can impact the condition of patients at the end of life, particularly in the face of the current challenges posed by climate change. We obtained temperature data for Northern Taiwan from a public data set as a new input feature and examined their effect on model performance [56]. However, owing to limitations in interpretation and the fact that this feature was not originally included in our study design, we have included the analysis results in Multimedia Appendix 6. Nevertheless, we believe that the inclusion of environmental factors in prediction models holds promise, provided that it is done within the framework of a well-designed study and a larger cohort in the future.

### Limitations

Our study is subject to several limitations that should be acknowledged. First, there was a large variation in survival time among the patients, resulting in data imbalances within our data set. We attempted to mitigate this issue by performing downsampling on individuals with longer survival times. Second, the nature of our study resulted in a class imbalance between the positive and negative labels. We intentionally did not perform any processing on the testing set to maintain the real-world distribution of the data. We believe that the performance of the models can be adequately evaluated using precision, recall, and *F*_1_-score. Third, it is important to note that our study was conducted on a relatively small cohort. Therefore, further validation is necessary to ensure the robustness of the prediction models.

### Future Scope of Wearable Devices and AI in End-of-Life Care

Many patients with terminal cancer expressed a preference for living and dying at home, as it provides comfort, autonomy, security, and social interaction [19-22,57]. High-quality home-based and outpatient end-of-life care relies on timely medical and emotional support from the care team and good preparedness for death [23,58]. This study presents a prototype that demonstrates the potential of combining wearable devices and machine learning models to perform noninvasive, real-time monitoring of physical conditions and provide an early warning system for impending death events. To further advance this field, we propose three key directions for future research:

1. External validation: validate the robustness of prediction models in different health care facilities and among diverse populations, including patients at the end of life without a cancer diagnosis, to ensure the generalizability of the models.
2. Prediction of other important events: explore the prediction of crucial events other than death in end-of-life care, such as emergent medical needs that require timely intervention. These predictions can significantly impact the quality of care provided to home-based patients.
3. Real-world application: assess the benefits of integrating wearable devices and AI into real-world end-of-life care settings. For example, determine whether AI-powered predictions can help reduce emergency department visits or whether wearable devices enhance patients’ sense of safety during home care. Further research is needed to examine the practical application of wearable devices and AI in end-of-life care, with a focus on improving the overall quality of the care provided.

### Conclusions

The findings of this study suggest that it is possible to predict death events among patients with terminal cancer using wearable devices and machine learning techniques. Although our prototype demonstrates the potential of these approaches in end-of-life care, further research is needed to confirm the robustness of the models and their effectiveness in real-world settings.

## Appendix

Multimedia Appendix 1 Survival prediction tools in end-of-life care.

Multimedia Appendix 2 Clinical assessment items (translated to English).

Multimedia Appendix 3 Shapley additive explanations value analysis of different models (extreme gradient boosting, deep neural network, random forest, and k-nearest neighbor).

Multimedia Appendix 4 Shapley additive explanations value analysis of the wrong prediction cases (extreme gradient boosting).

Multimedia Appendix 5 The extreme gradient boosting model’s predictions in different age groups.

Multimedia Appendix 6 Using environmental factors in predicting the end-of-life status.

## Abbreviations

AdaBoost: adaptive boosting
AI: artificial intelligence
AKPS: Australia-modified Karnofsky Performance Status
AUROC: area under the receiver operating characteristic curve
HR: heart rate
KNN: k-nearest neighbor
SHAP: Shapley additive explanations
SpO2: blood oxygen saturation
XGBoost: extreme gradient boosting

## Data Availability

Deidentified data and a data dictionary supporting the findings of this study will be made available upon request to researchers who submit a methodologically sound proposal and have obtained approval from the study authors. The data are not publicly available because of the limited consent obtained from the participants, who agreed to use the data only for research in palliative medicine or end-of-life care. The data will be made available immediately following publication, and we guarantee its availability until at least December 31, 2027, with no anticipated end date. Researchers interested in accessing the data should submit their proposals to JST on his email ID.
