# Supplementary material for: Evaluating the Potential of Machine Learning and Wearable Devices in End-of-Life Care in Predicting 7-Day Death Events Among Patients With Terminal Cancer: Cohort Study
Source: J Med Internet Res. 2023 Aug 18;25:e47366. doi: 10.2196/47366 (PMC10474512; doi:10.2196/47366)
Supplement: Multimedia Appendix 1 [file jmir_v25i1e47366_app1.pdf]

Appendix 1. Survival prediction tools in end-of-life care

Over the past few decades, numerous clinical tools for survival prediction have been developed and validated through extensive research. Some of the most well-known and widely used tools are described below, highlighting their key features and limitations.

Palliative Performance Scale (PPS)

The Palliative Performance Scale (PPS) was first developed in 1996 and is based on a patient's level of functioning in areas such as "ambulation," "activity and evidence of disease," "self-care," "intake," and "consciousness." It divides patients into 11 levels, ranging from 0 to 100 [1]. This tool has been conducted in palliative care settings, where the majority of patients have a life expectancy of less than 30 days, and it was found that patients with lower PPS scores had a shorter average survival time [3]. Survival estimates ranged from 1 to 3 days for patients with PPS scores of 10% compared with 5 to 36 days, which is a relatively wider range, for those with scores of 30% [4]. In nursing homes and community settings, there were also statistically significant differences in survival rates among patients classified by PPS scores over 3-6 months, indicating this tool is applicable to both cancer and non-cancer patients [2].

Palliative Prognostic Score (PaP)

In Palliative Prognostic Score (PaP), patients are categorized into three groups

based on scores derived from factors such as "dyspnea," "anorexia," "Karnofsky Performance Status (KPS)," "white blood cell (WBC) count," and "lymphocyte ratio," along with healthcare professionals' "clinical prediction of survival" [9]. This categorization can effectively predict 30-day survival rates [10,11]. The Palliative Prognostic Score (PaP) was originally developed for patients with advanced cancer receiving palliative care, but subsequent research has shown that it can effectively differentiate non-cancer patients as well [12]. However, the use of PaP can be demanding, as it relies on clinical judgment by healthcare professionals and requires recent blood test data, which is not regularly checked in terminal care.

Palliative Prognostic Index (PPI)

The Palliative Prognostic Index (PPI) serves as a comprehensive tool by incorporating various clinical assessments, including the Palliative Performance Scale (PPS), oral intake, the presence of edema, dyspnea, and delirium [5]. These parameters play a crucial role in evaluating patients' overall condition and predicting their survival outcomes. By utilizing the PPI index, healthcare professionals can stratify patients into three distinct groups, allowing for reliable predictions of their survival rates at both 3 weeks and 6 weeks [6,7]. Furthermore, the PPI index has also been utilized in another study to predict life expectancy of less than 30 days, emphasizing its versatility and applicability in different timeframes [8]. Overall, the PPI index has proven to be a

valuable and effective method in predicting survival length in a range of 3 to 6 weeks.

Glasgow Prognostic Score (GPS)

The Glasgow Prognostic Score (GPS) is solely based on blood test values of CRP (C-reactive protein) and albumin for prognostic assessment. These biomarkers are indicative of the body's inflammatory state and offer valuable insights into the severity of the disease, making them effective substitutes for clinical experience or tumor grading in the evaluation process [13,14]. However, it is important to note that the GPS is primarily designed for assessing longer-term survival estimates, typically spanning several months. To obtain accurate evaluations using the GPS, blood tests are required to measure CRP and albumin levels.

Other Tools

Many of the tools mentioned above were initially developed during the late 1990s and early 2000s. However, recent studies have further explored the relationship between commonly used clinical functional tests, such as walking, eating, and communication, and their correlation with previous scales. These investigations have contributed to the advancement of purely functional prognostic assessments, which provide valuable insights into the prognosis of patients [15]. In Taiwan, there have been notable studies that have consolidated the link between commonly used assessments, various symptoms, and prognostic outcomes [16]. Together, these findings highlight the

ongoing research efforts aimed at enhancing the accuracy and applicability of prognostic evaluations in clinical settings.
