# Supplementary material for: Evaluating the Potential of Machine Learning and Wearable Devices in End-of-Life Care in Predicting 7-Day Death Events Among Patients With Terminal Cancer: Cohort Study
Source: J Med Internet Res. 2023 Aug 18;25:e47366. doi: 10.2196/47366 (PMC10474512; doi:10.2196/47366)
Supplement: Multimedia Appendix 2 [file jmir_v25i1e47366_app2.pdf]

Appendix 2. Clinical assessment items (translated into English)

Item	Question	Value
Consciousness	What is the patient's consciousness status now and during the past 7 days?	1- Clear<br>2- On and off, with occasional disturbance or delirium<br>3- Mostly drowsiness, can be waken up for a very short time<br>4- Totally comatose.
Appetite	What is the patient's intake status now and during the past 7 days?	1- Normal intake amount<br>2- Reduced (at least mouthfuls)<br>3- Minimal (< 1-2 mouthfuls)<br>4- No oral intake
Urination	What is the patient's urine output status now and during the past 7 days?	1- Normal<br>2- Reduced<br>3- None / Minimal
IV/SC Fluid use	Is the patient receiving any fluid therapy through intravenous (IV) or subcutaneous (SC) route?	0- No<br>1- Yes (normal saline, glucose, etc.)<br>2- Yes (Total parenteral nutrition)
Edema	Does the patient have edema in any body part?	0- No<br>1- Yes
Pressure sore	Does the patient have pressure in any body part?	0- No<br>1- Yes
Pain scale	How painful are you?<br>How painful is the patient do you think? (if answered by caregiver)	0 (None) – 10 (Most painful)
Sleep	How is the sleep quality in the past 7 days?	1- Normal / Good<br>2- Mild sleep disturbance (wake up sometimes)<br>3- Severe sleep disturbance (hard to fall asleep or wake up multiple times due to symptoms)<br>4- Drowsiness all the time
Nausea/Vomiting	Does the patient suffer from nausea or vomiting in the past 7 days?	1- No<br>2- Mild (Occasional)<br>3- Severe (Often/Always)
Constipation	Does the patient suffer from constipation in the past 7 days?	1- No<br>2- Mild (3-4 days)<br>3- Severe (> 5 days)

Diarrhea	Does the patient suffer from diarrhea in the past 7 days?	1- No<br>2- Mild (3-4 days)<br>3- Severe (> 5 days)
Dyspnea	Does the patient feel shortness of breath?	1- No<br>2- Mild (Only when moving or specific posture)<br>3- Severe (Always, include resting)
Fatigue	Generally speaking, does the patient feel fatigue?	1- No, activities as usual<br>2- Mild (Still do some activities, but need more rest)<br>3- Severe (Resting most of the time)
Anxiety	Do you feel anxious?<br>Is the patient anxious? (if answered by caregivers)	0 (None) – 10 (Most anxious)
Depression	Do you feel depressed?<br>Is the patient depressed? (if answered by caregivers)	0 (None) – 10 (Most depressed)
Functional level	What is the functional level of patient assessed in Australia-modified Karnofsky Performance Status (AKPS)?[a]	100 (normal) – 0 (dead)
Clinical care phase	Which phase do you think the patient is in, according to the phase of illness in palliative care? [b]	1- Stable<br>2- Unstable<br>3- Deteriorating<br>4- Deceased

[a]Functional level is evaluated through AKPS, defined from the original work by Abernethy et al.[44]

[b]The question and options for the evaluation of clinical care phase is according to the work by Masso et al.[45]
