# Supplementary material for: Evaluating the Potential of Machine Learning and Wearable Devices in End-of-Life Care in Predicting 7-Day Death Events Among Patients With Terminal Cancer: Cohort Study
Source: J Med Internet Res. 2023 Aug 18;25:e47366. doi: 10.2196/47366 (PMC10474512; doi:10.2196/47366)

Appendix 3. The SHAP value analysis of different models

SHAP summary plot (XGBoost)

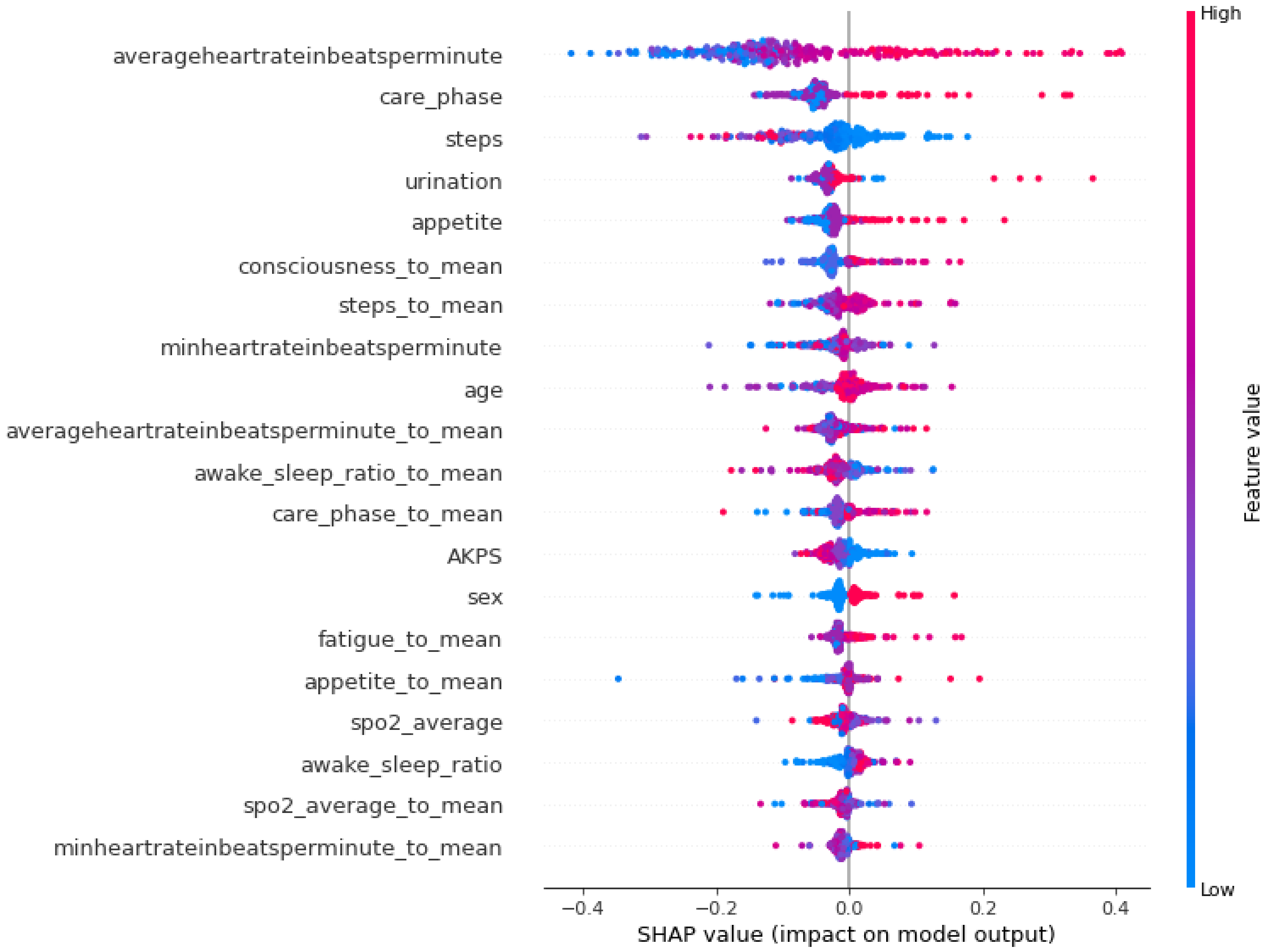

Mean absolute SHAP value (XGBoost)

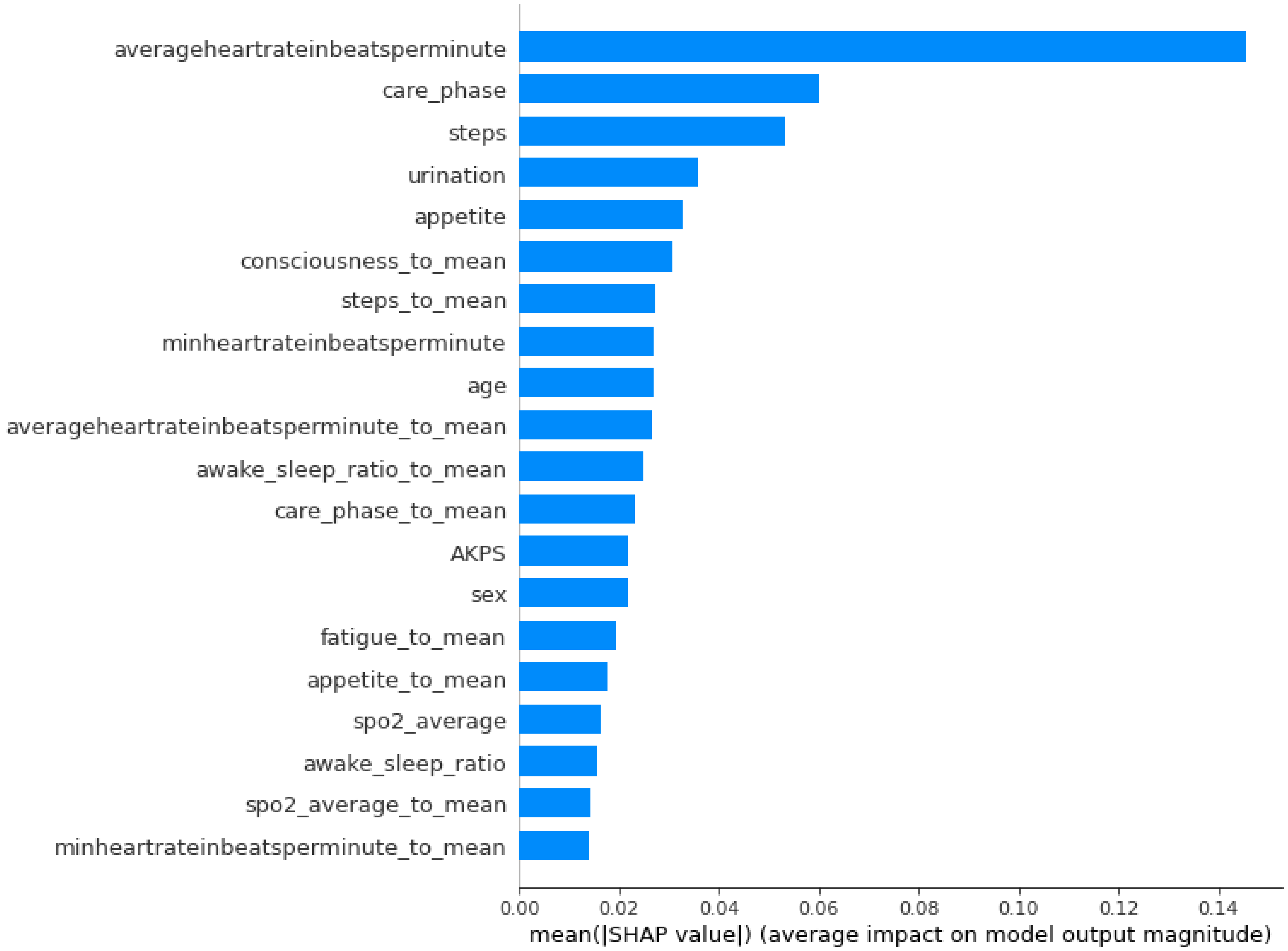

SHAP summary plot (DNN)

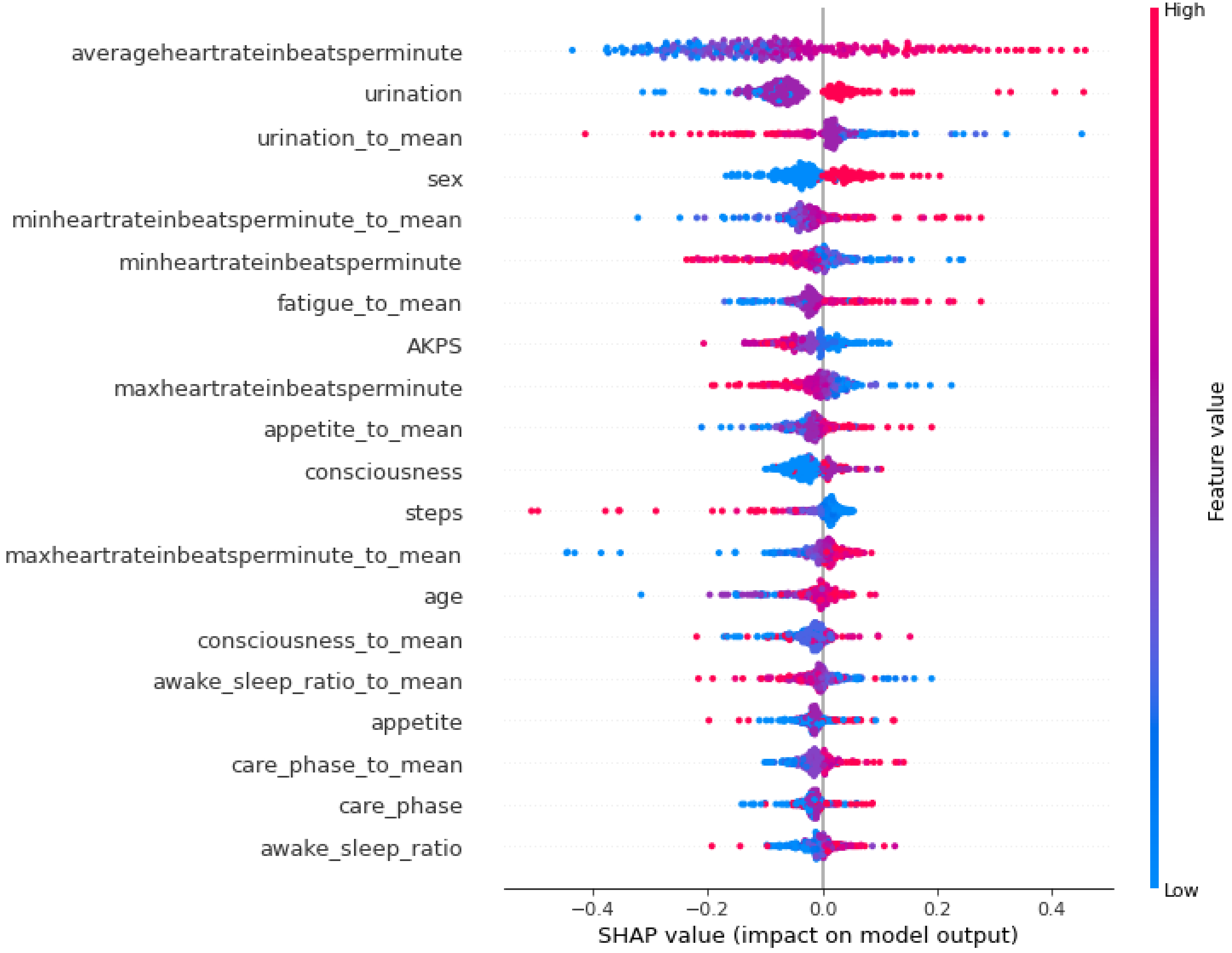

Mean absolute SHAP value (DNN)

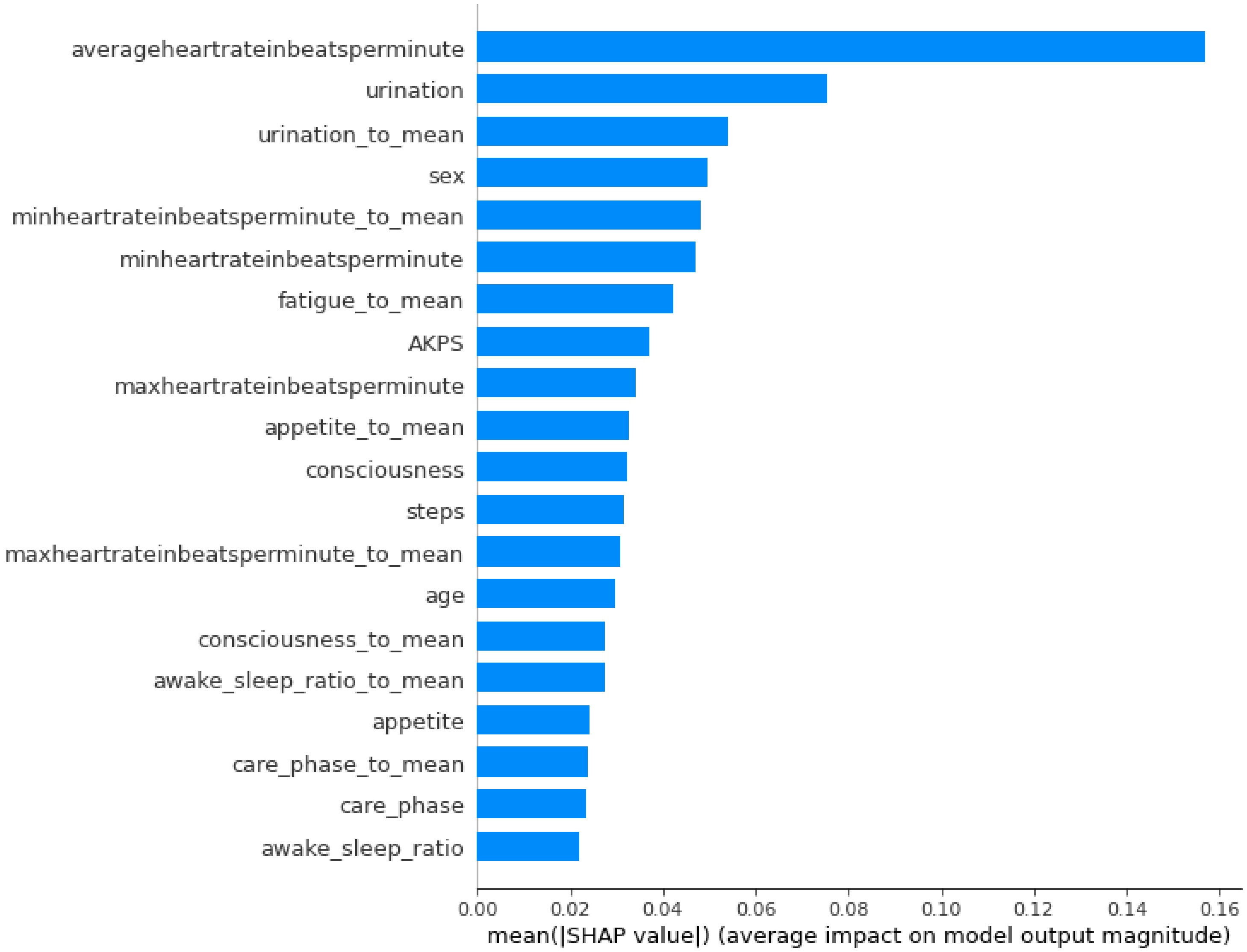

SHAP summary plot (RF)

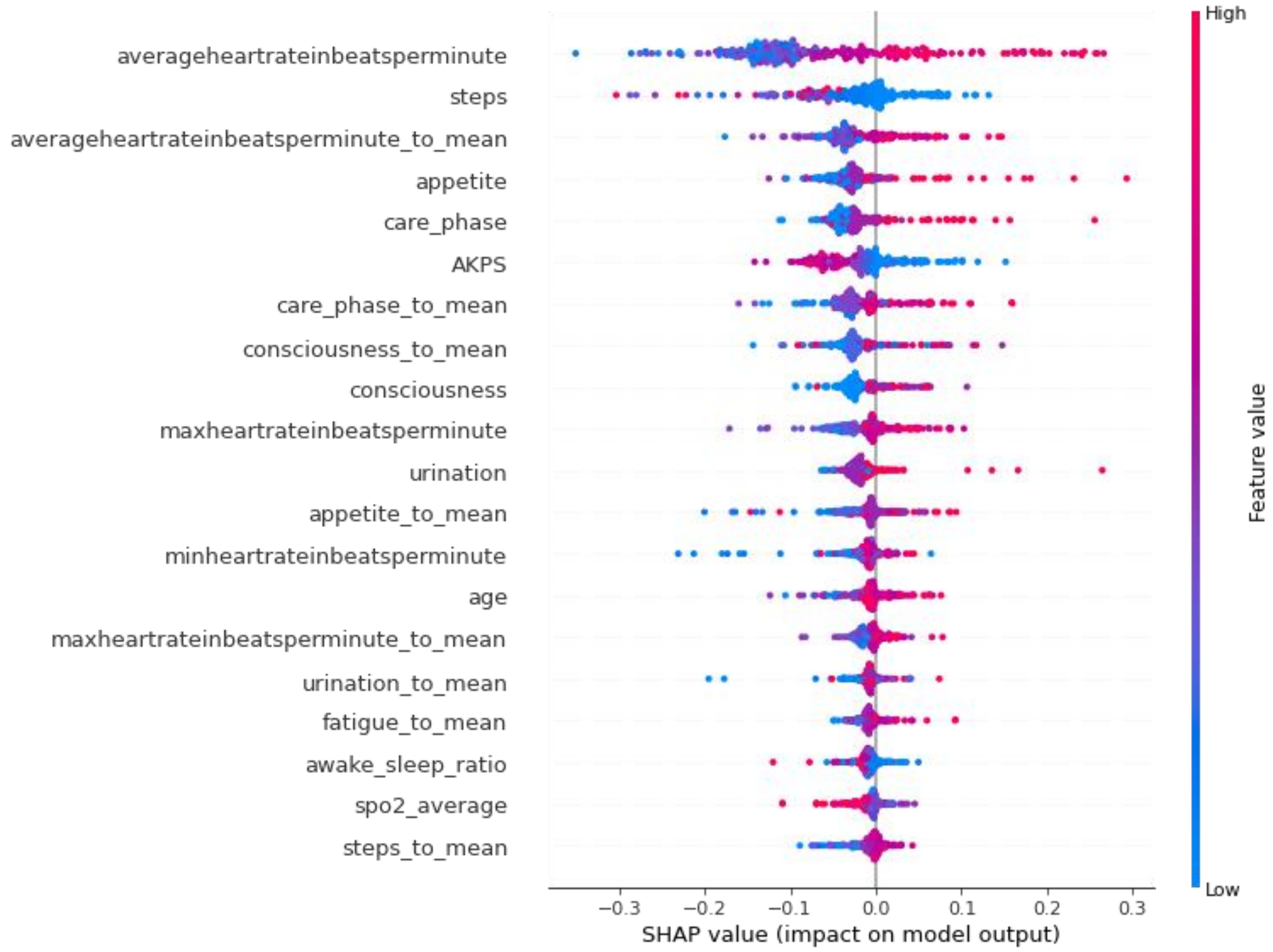

Mean absolute SHAP value (RF)

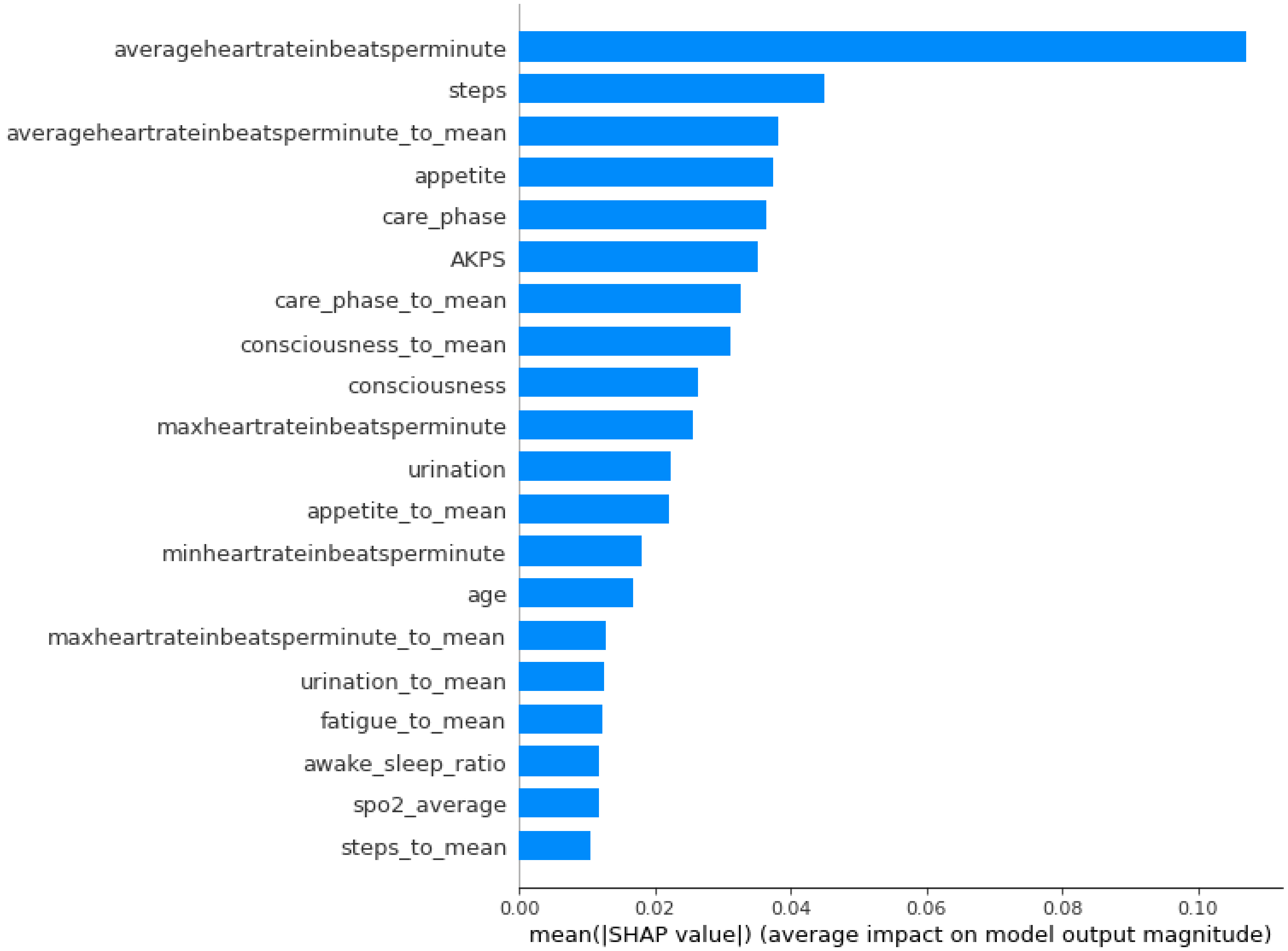

SHAP summary plot (KNN)

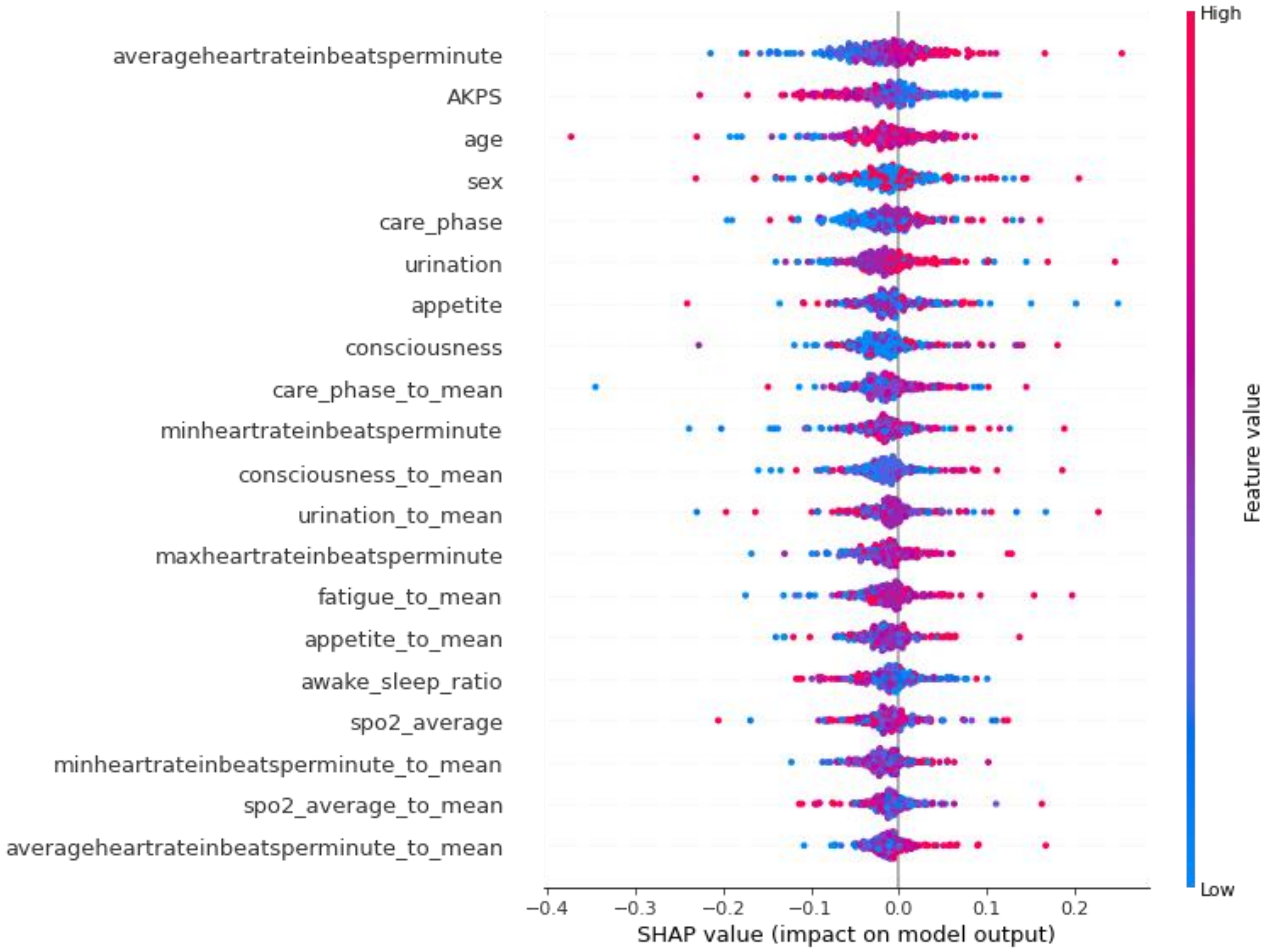

Mean absolute SHAP value (KNN)

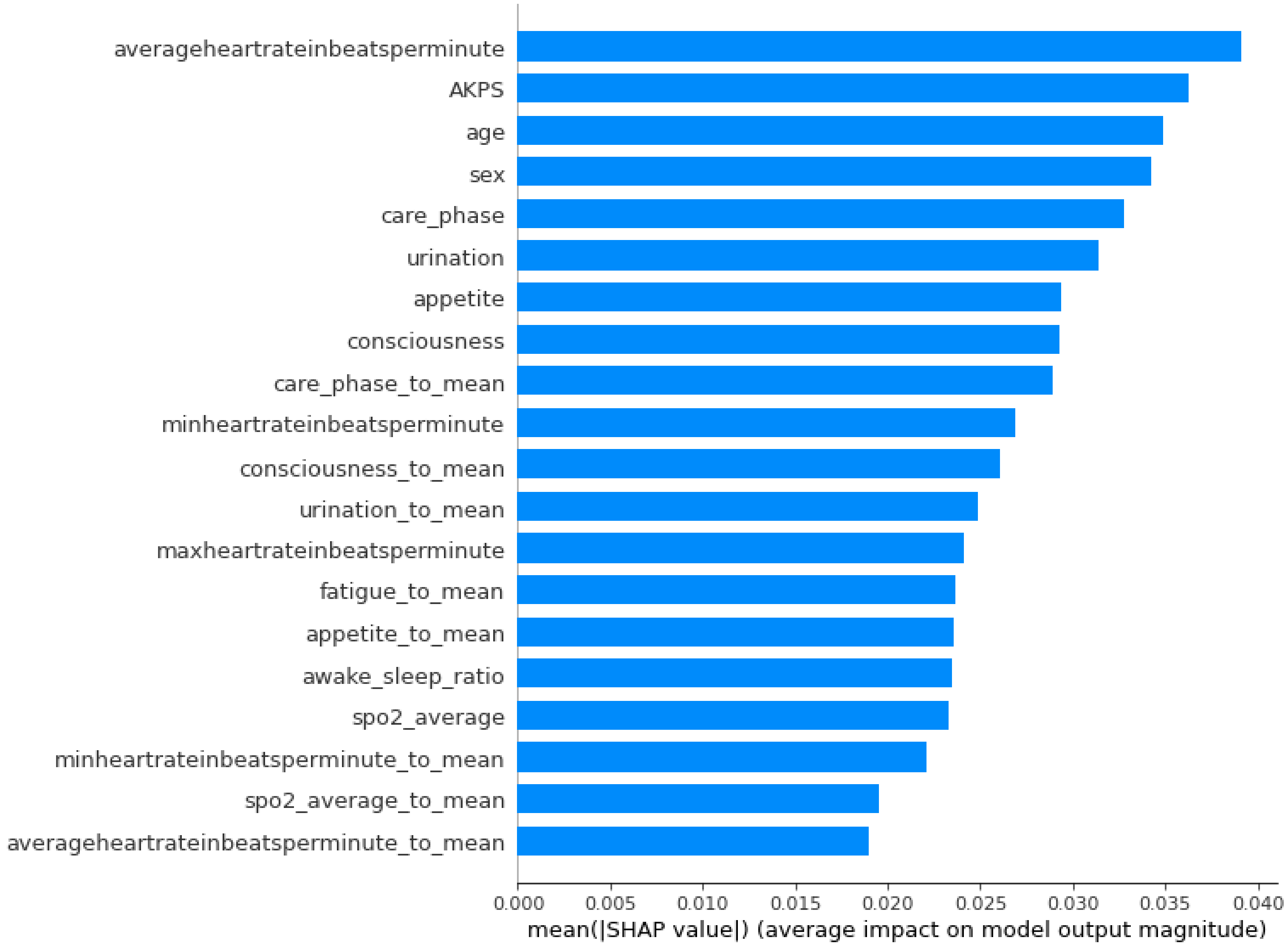

Supplement: Multimedia Appendix 3 [file jmir_v25i1e47366_app3.pdf]
