# Supplementary material for: Evaluating the Potential of Machine Learning and Wearable Devices in End-of-Life Care in Predicting 7-Day Death Events Among Patients With Terminal Cancer: Cohort Study
Source: J Med Internet Res. 2023 Aug 18;25:e47366. doi: 10.2196/47366 (PMC10474512; doi:10.2196/47366)

Appendix 4. The SHAP value analysis of wrong prediction cases.

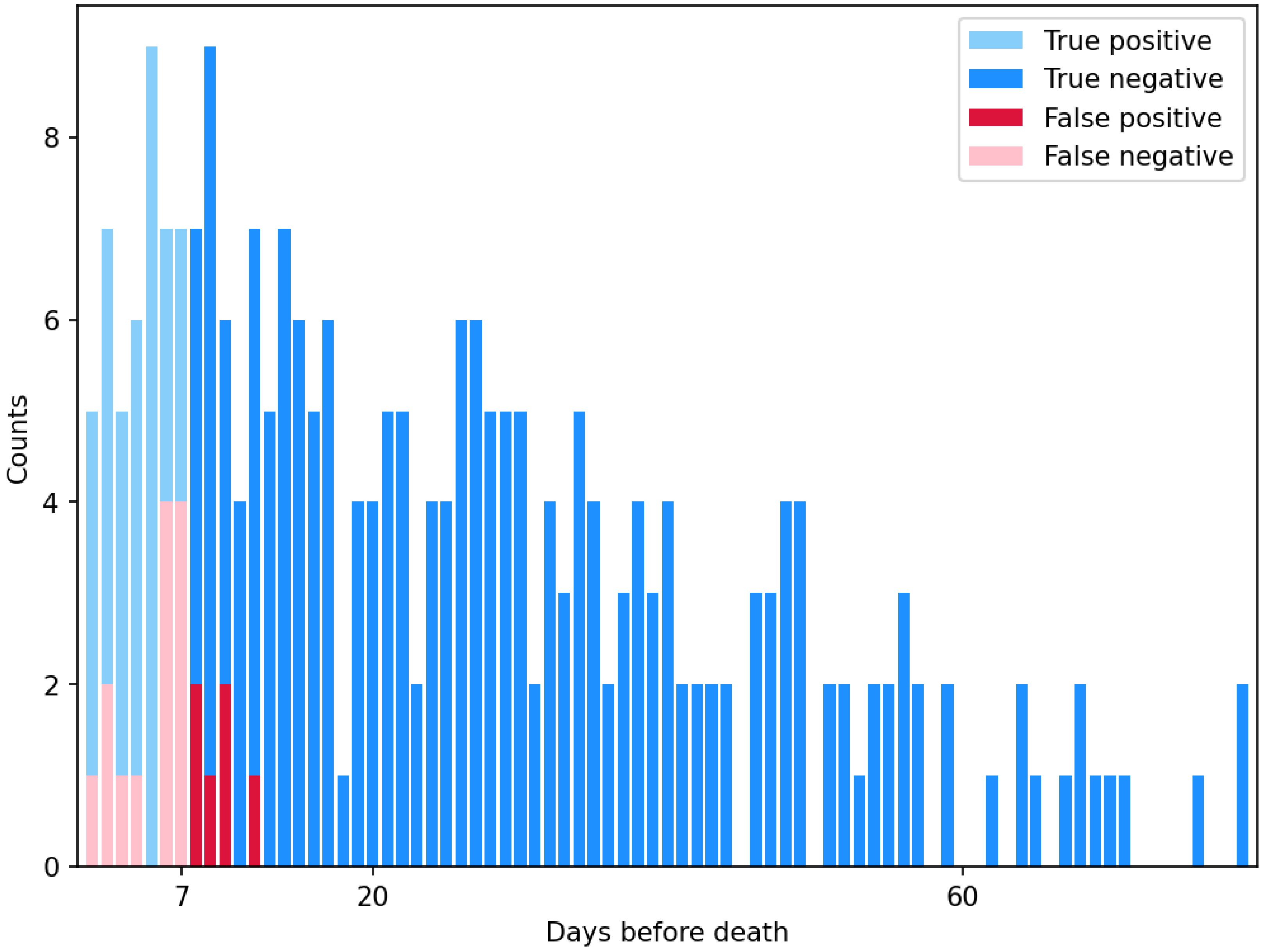

SHAP value analysis of XGBoost : **False positive** (8-12 days before death)

Average_HR → positive effect to prediction

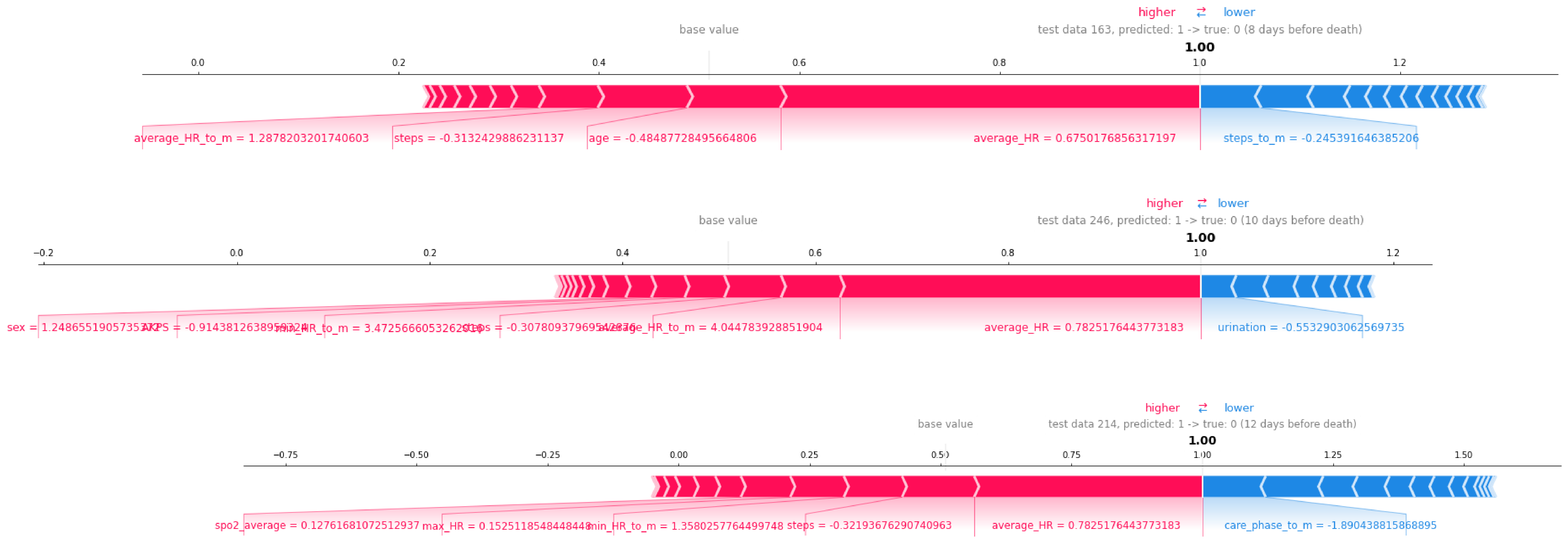

SHAP value analysis of XGBoost : **False positive** (8-12 days before death)

Average_HR → minimal effect to prediction

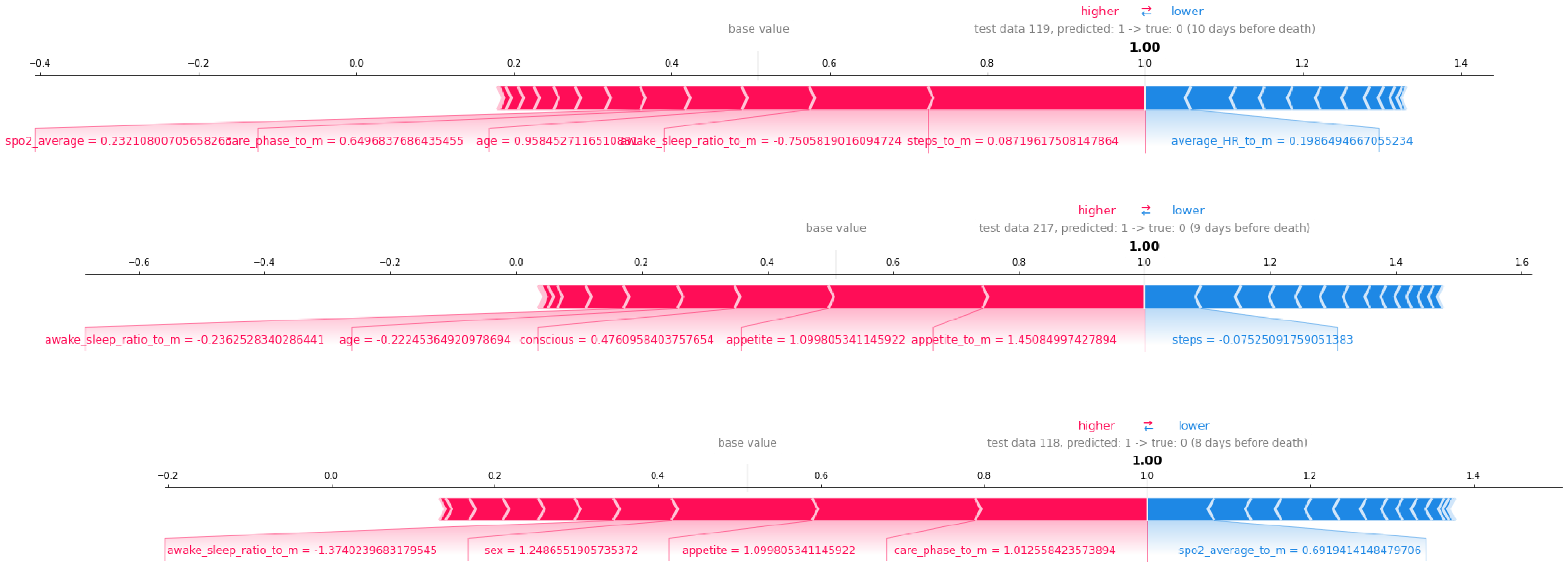

SHAP value analysis of XGBoost : **False negative** (6-7 days before death)

Average_HR → negative effect to prediction

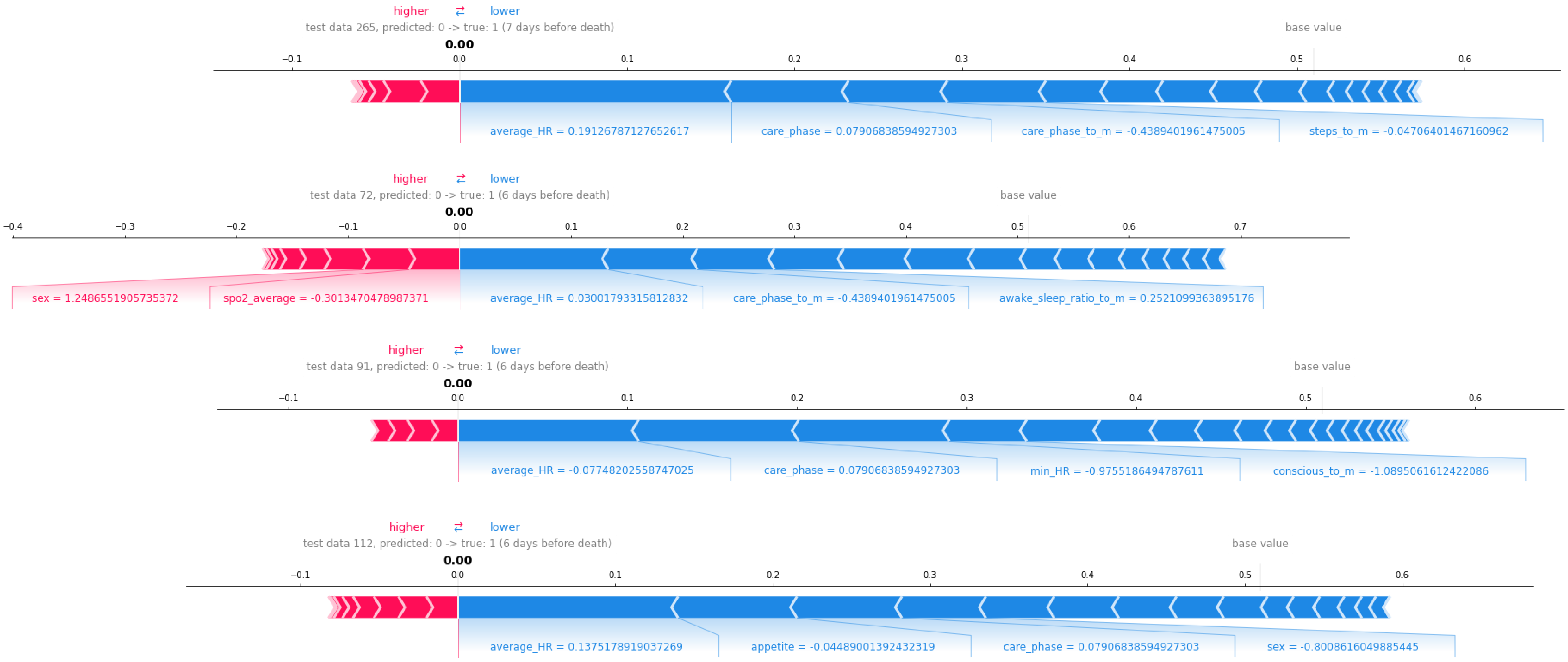

SHAP value analysis of XGBoost : **False negative** (6-7 days before death)

Average_HR → positive effect to prediction

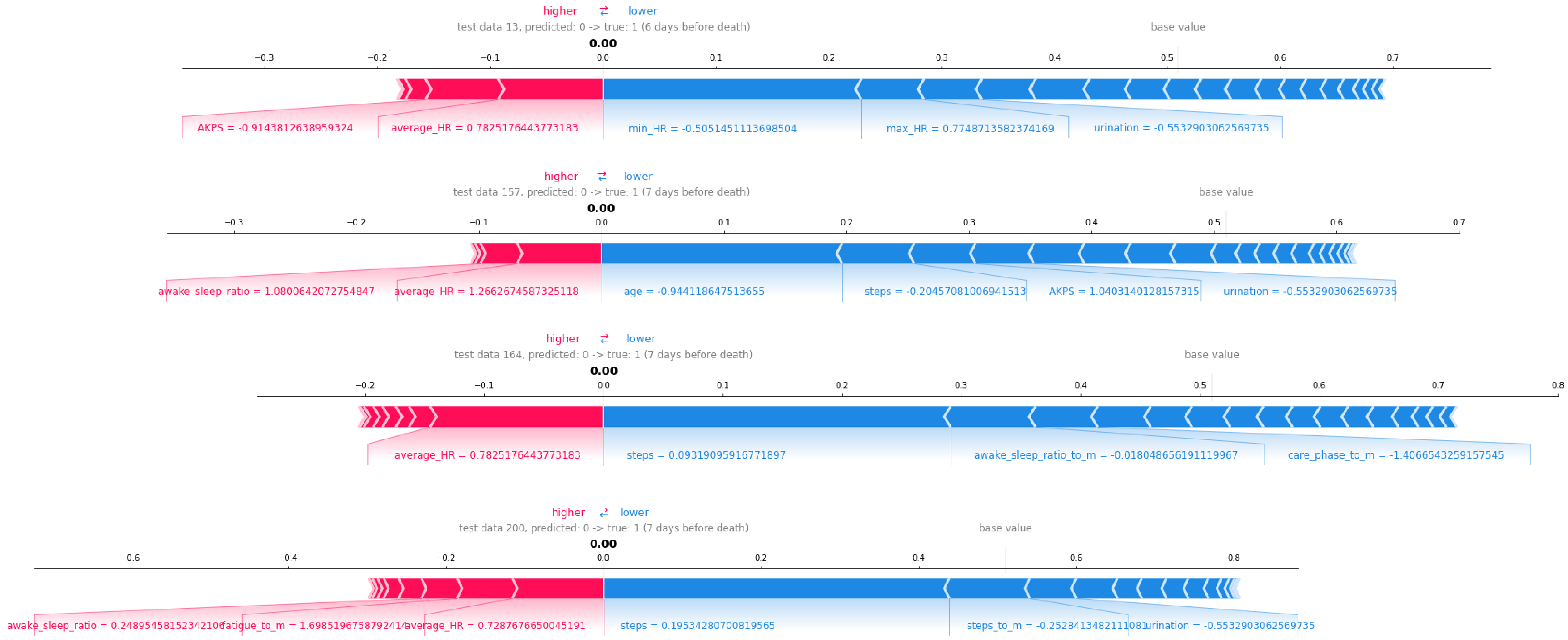

SHAP value analysis of XGBoost : **False negative** (1-4 days before death)

Average_HR → negative effect to prediction

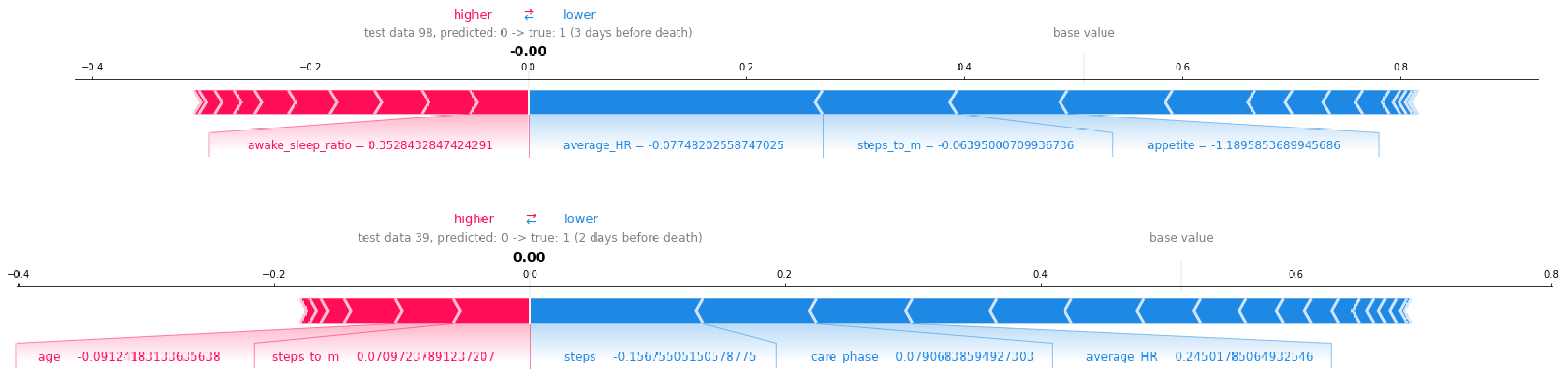

SHAP value analysis of XGBoost : **False negative** (1-4 days before death)

Average_HR → positive effect to prediction

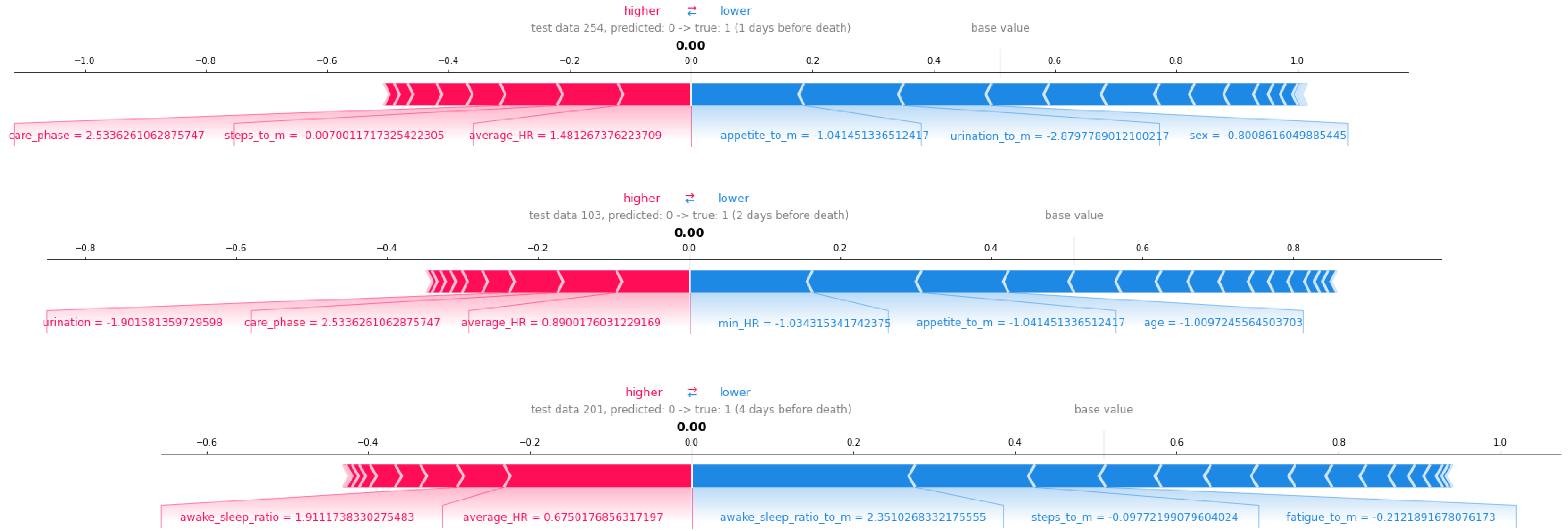

Supplement: Multimedia Appendix 4 [file jmir_v25i1e47366_app4.pdf]
