# Supplementary material for: Evaluating the Potential of Machine Learning and Wearable Devices in End-of-Life Care in Predicting 7-Day Death Events Among Patients With Terminal Cancer: Cohort Study
Source: J Med Internet Res. 2023 Aug 18;25:e47366. doi: 10.2196/47366 (PMC10474512; doi:10.2196/47366)

Appendix 5. The XGBoost Model Predictions in Different Age Group

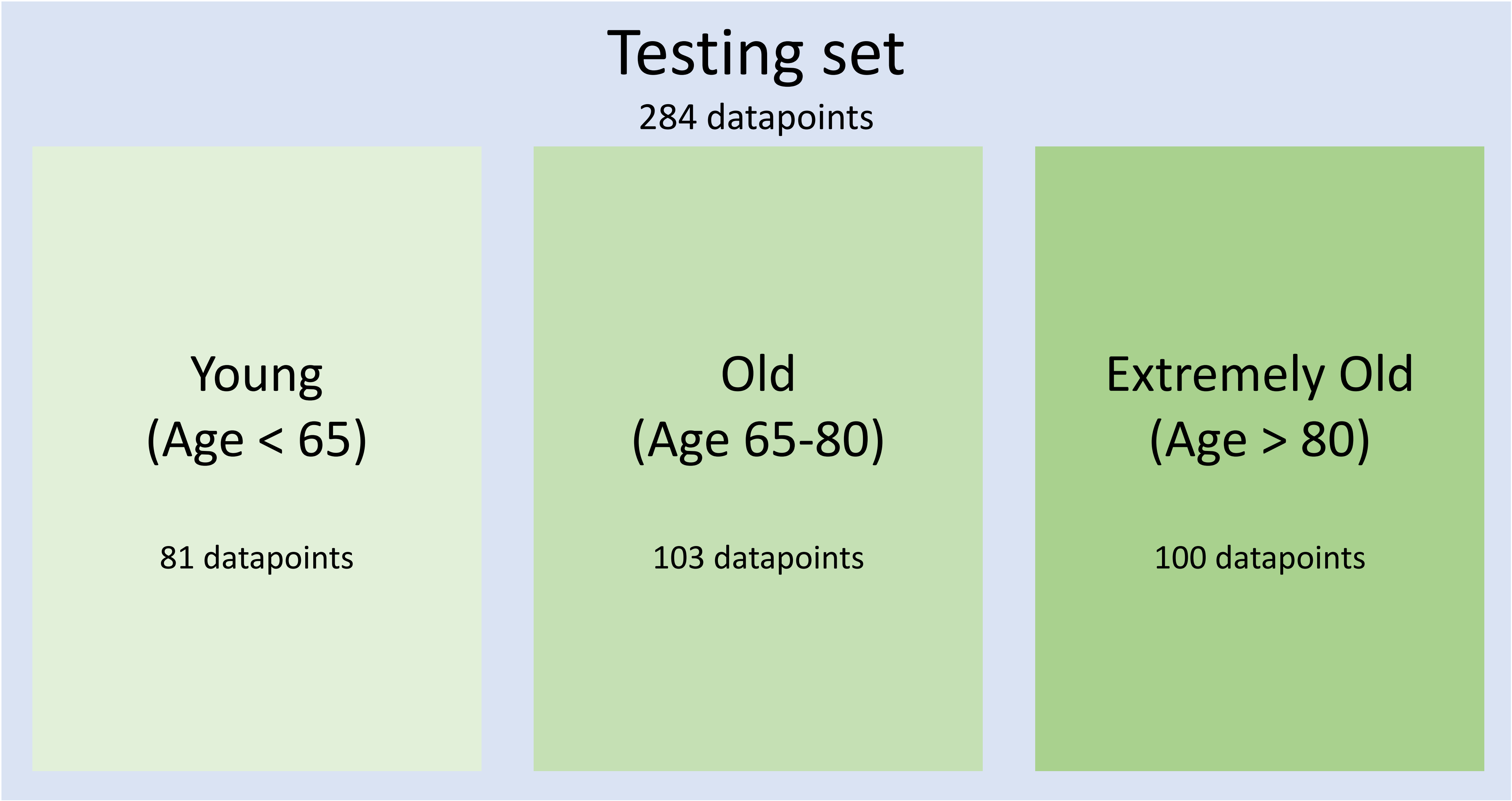

XGBoost Shapley summary plot (All)

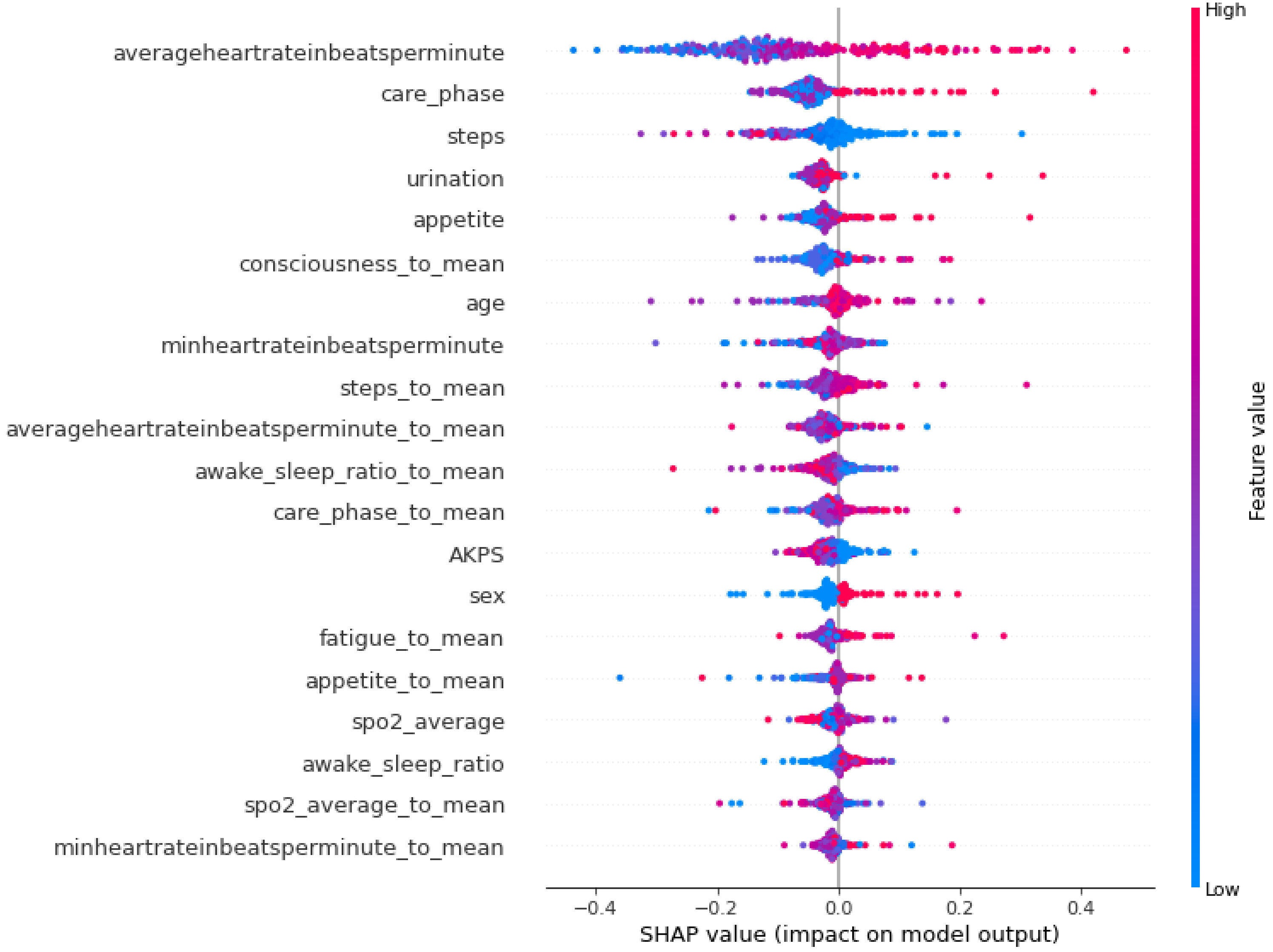

XGBoost Shapley summary plot (Young)

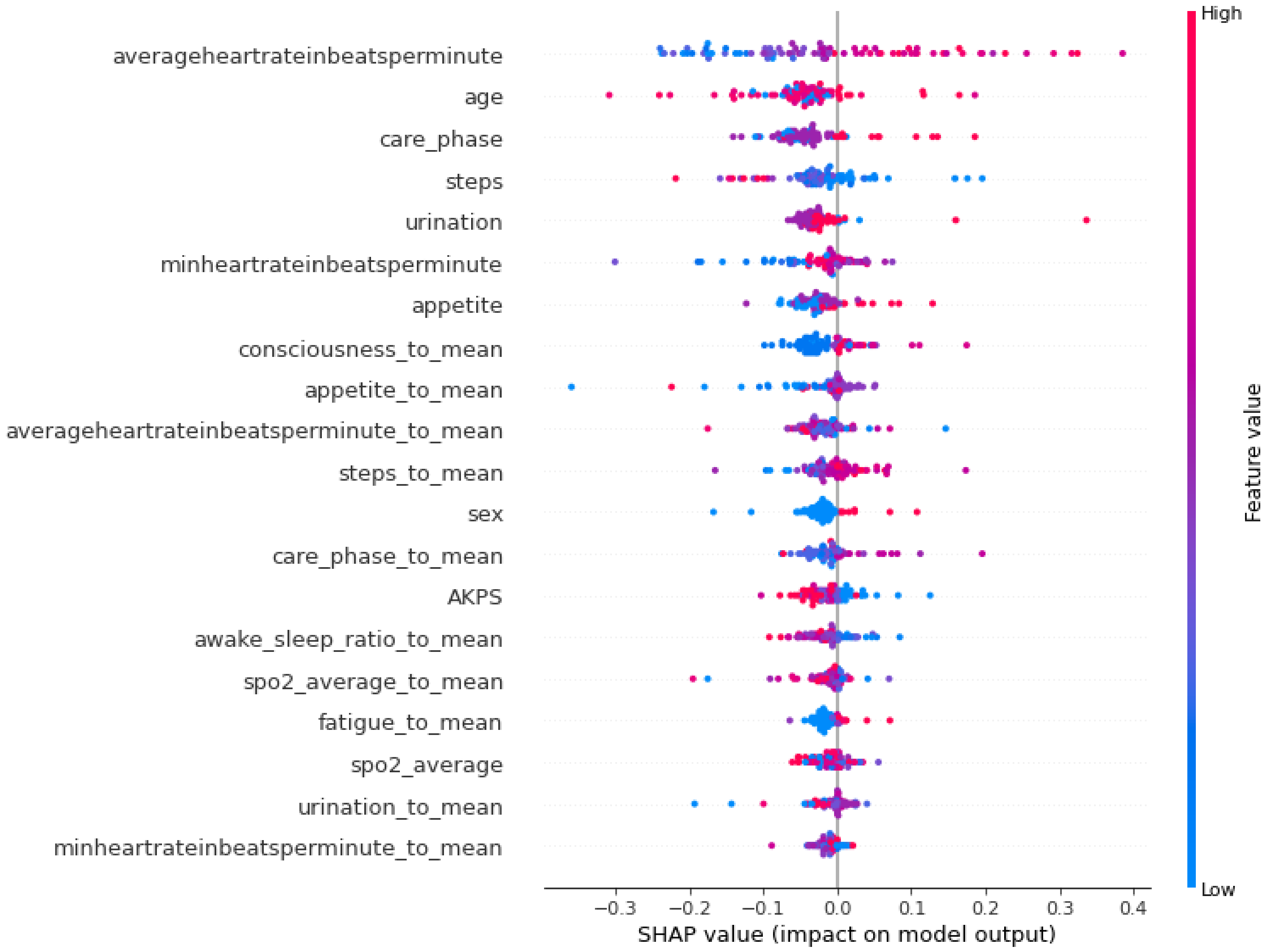

XGBoost Shapley summary plot (Old)

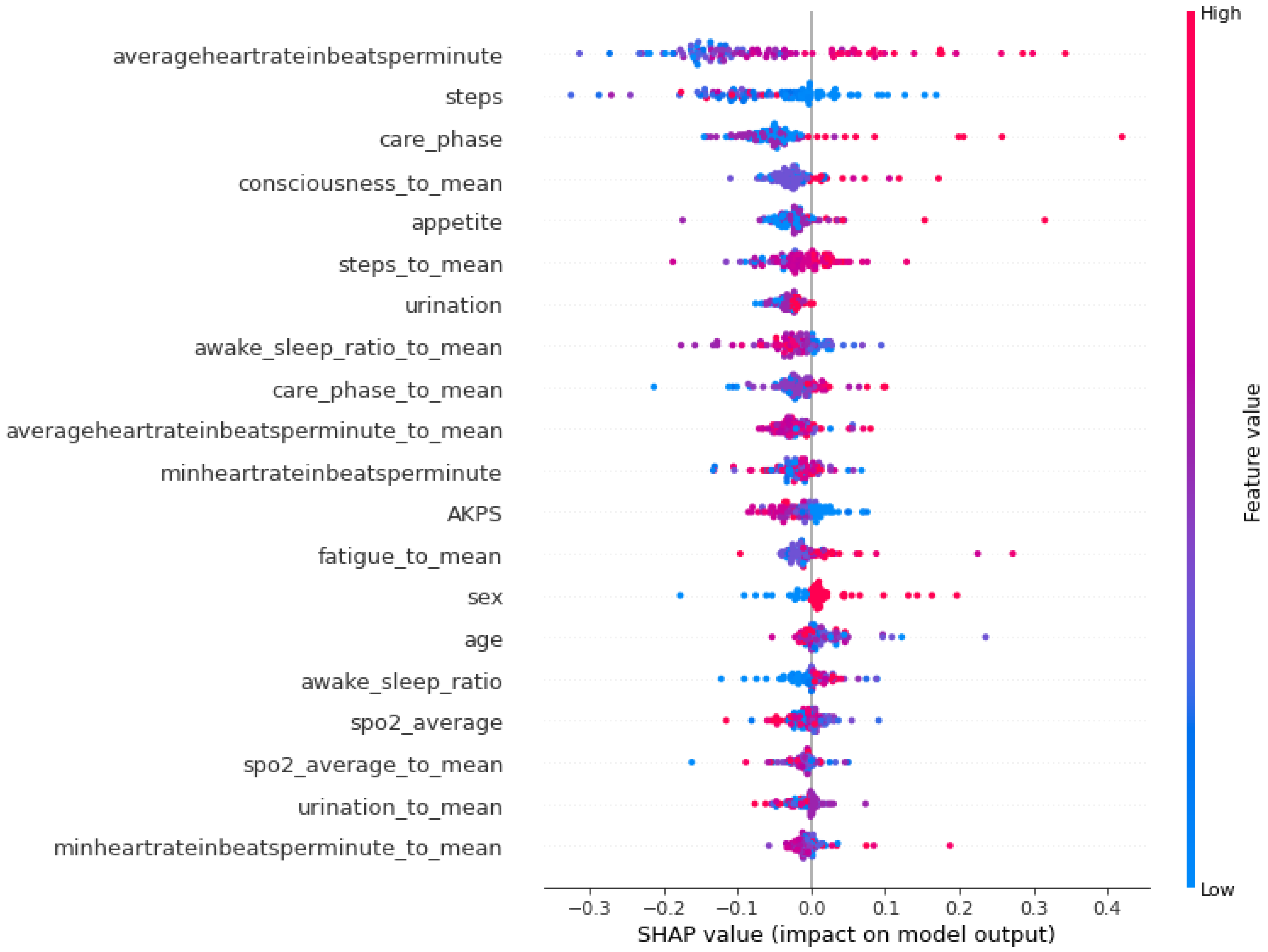

XGBoost Shapley summary plot (Extremely Old)

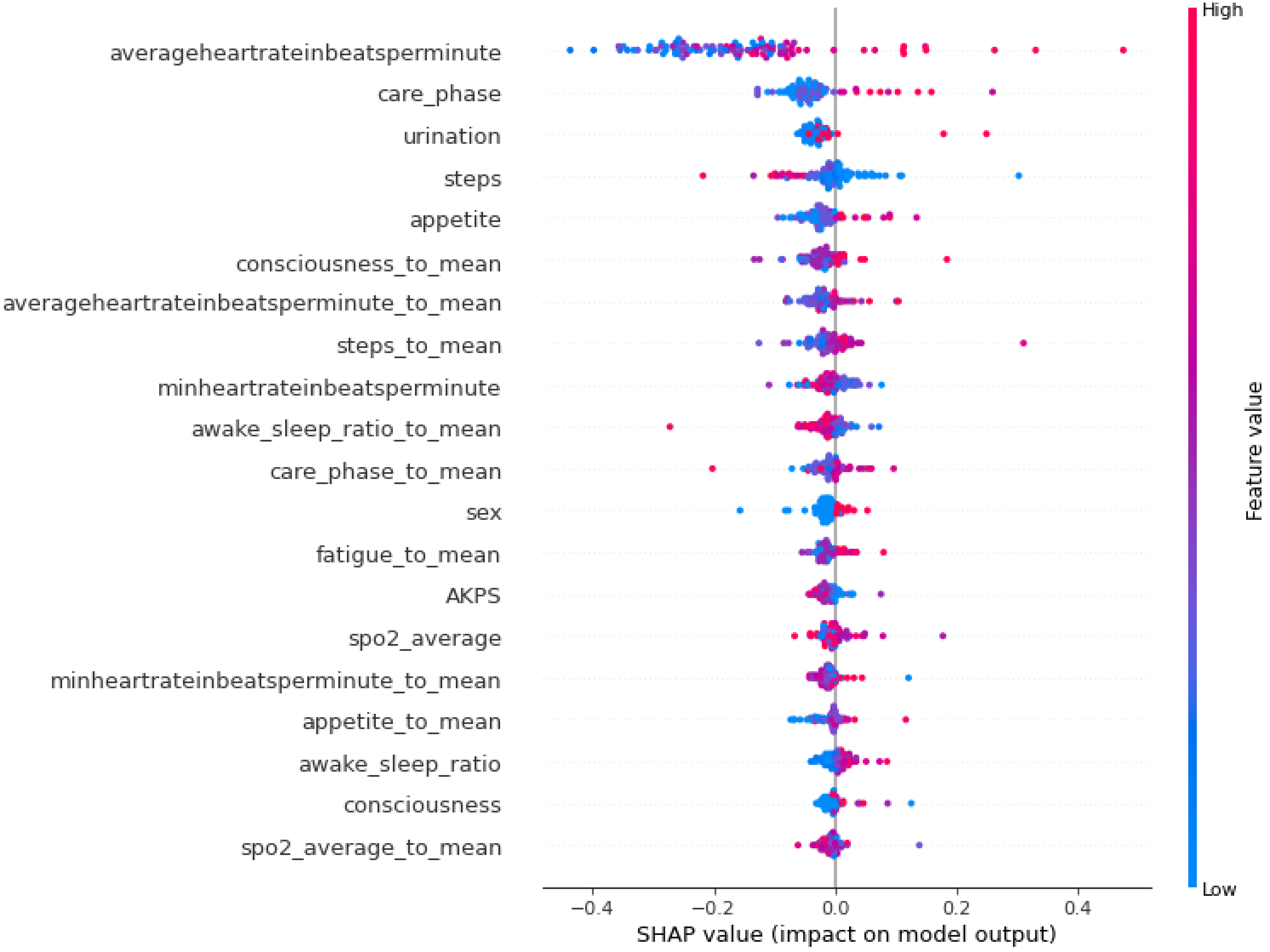

Shapley analysis of False Negative Cases (Young)

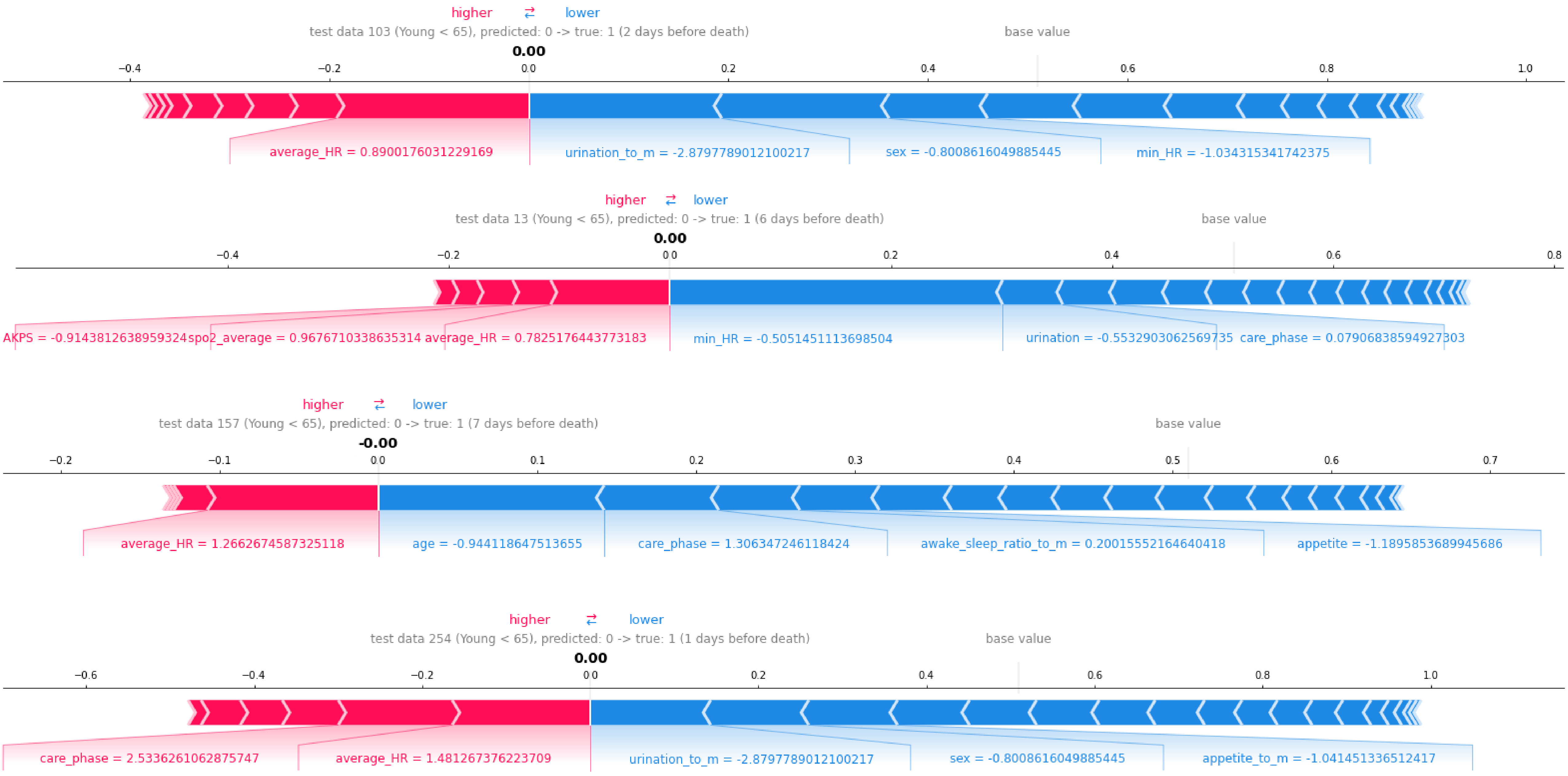

Shapley analysis of False Positive Cases (Young)

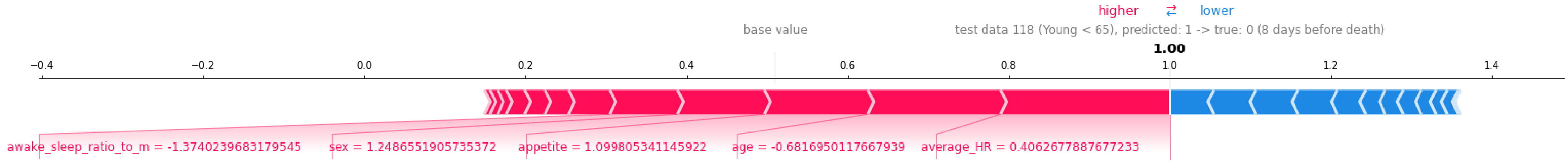

Shapley analysis of False Negative Cases (Extremely Old)

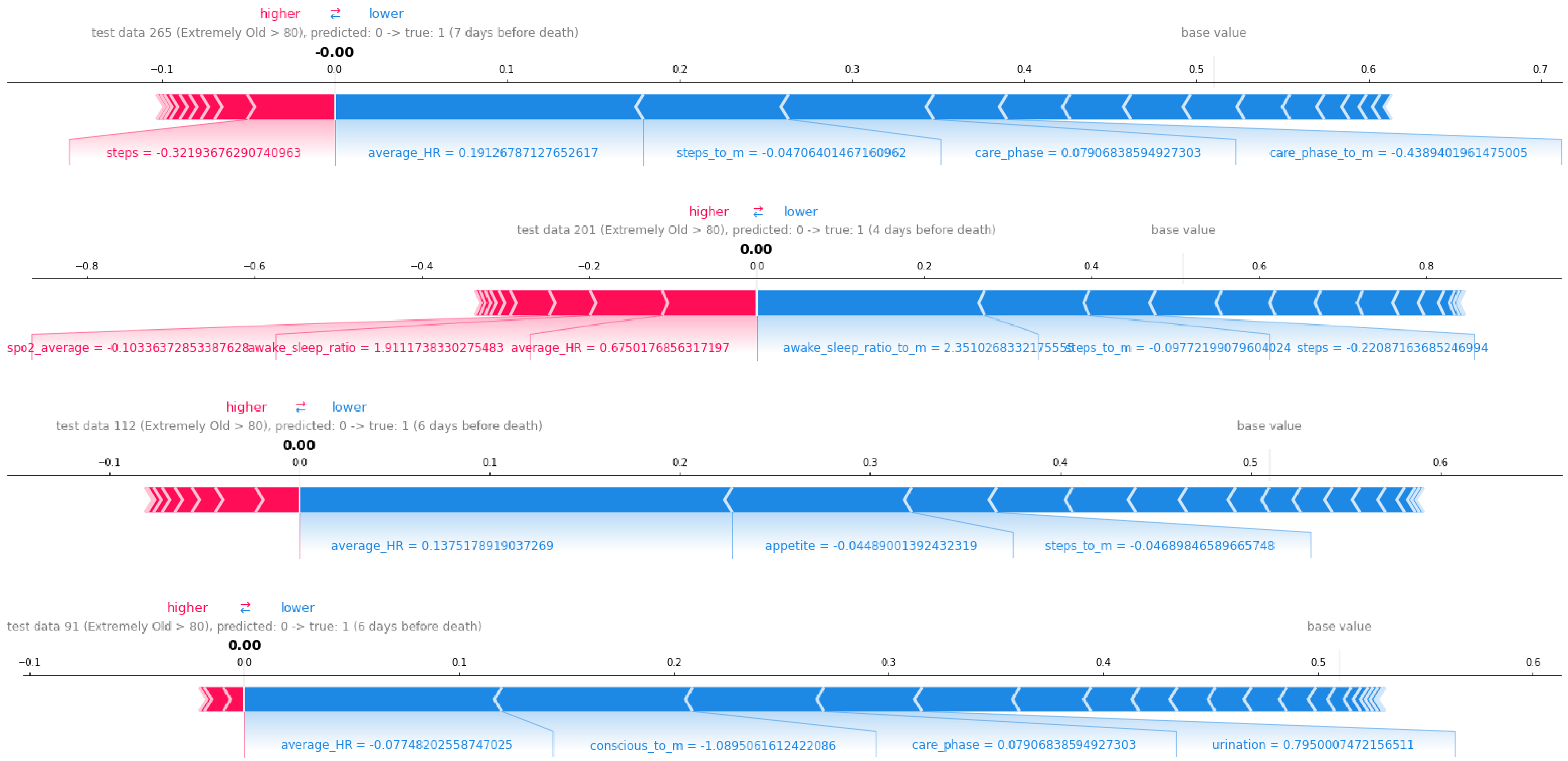

Shapley analysis of False Positive Cases (Extremely Old)

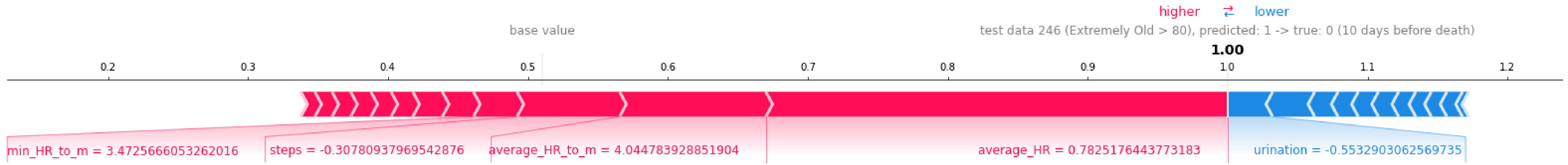

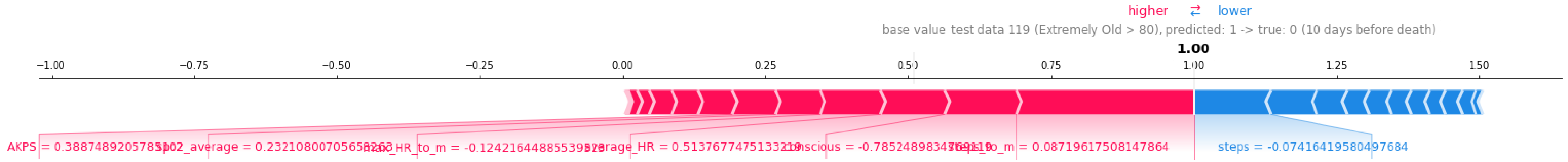

Supplement: Multimedia Appendix 5 [file jmir_v25i1e47366_app5.pdf]
