# Supplementary material for: Evaluating the Potential of Machine Learning and Wearable Devices in End-of-Life Care in Predicting 7-Day Death Events Among Patients With Terminal Cancer: Cohort Study
Source: J Med Internet Res. 2023 Aug 18;25:e47366. doi: 10.2196/47366 (PMC10474512; doi:10.2196/47366)
Supplement: Multimedia Appendix 6 [file jmir_v25i1e47366_app6.pdf]

Appendix 6. Adding Environmental Factors in Predicting End-of-life Status

It is well-known that extreme temperature events, such as sudden cold waves, can increase the risk of cardiac-related deaths, particularly in individuals with high cardiovascular risk and potentially end-of-life patients. Conversely, heatwaves during the summer months can contribute to heat-related deaths. However, the specific impact of changing temperatures on end-of-life patients remains uncertain.

While our study focused on using clinical assessment and wearable devices to predict end-of-life events in patients, we did not collect temperatures or other environmental factors in the original study design. To explore this issue, we included temperature as one of the features in our training dataset. We obtained temperature data for Taipei, Taiwan from the Central Weather Bureau of Taiwan (https://e-service.cwb.gov.tw/HistoryDataQuery/) and matched the mean temperature of each day to the corresponding data points. By adding average temperature as a feature and using the same hyperparameters as our original models, the XGBoost model achieved an F1 score of 0.82 and specificity of 0.98. In the Shapley analysis, "temperature" was ranked as the 9th important feature in the XGBoost model, with colder temperatures exerting a small but positive impact on the prediction of death events in most data points.

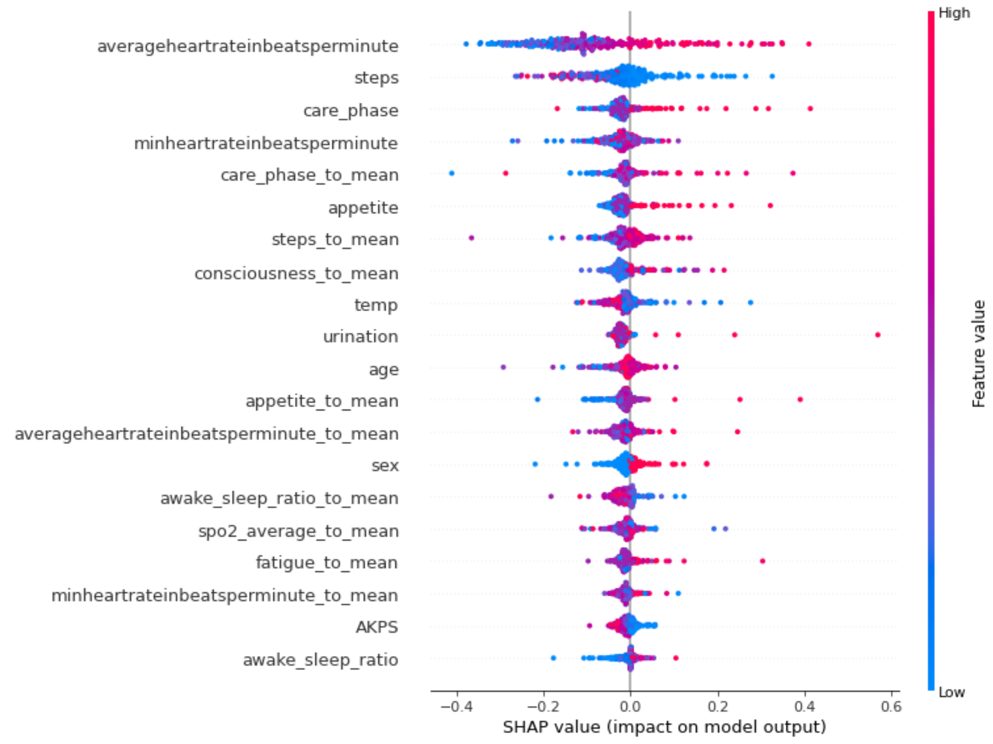

The Shapley summary plot of the XGBoost model with temperature as an input feature.

However, it is essential to acknowledge several limitations associated with this approach.

1. The temperature was measured at one specific meteorological monitoring station in Taipei. Although our patients primarily resided in Taipei and northern Taiwan, the exact location of each patient on any given day is unknown.
2. Our model does not account for indoor temperatures in the patients' living environments. It is worth noting that air conditioners are commonly installed and used due to Taiwan's subtropical climate. On the other hand, in our experiences, heaters are not commonly employed. Therefore, a more precise assessment of the impact would require direct measurement of the ambient temperature around the patient, which was not feasible in our study.
3. Within the context of climate change, we had anticipated exploring the impact of high temperatures on end-of-life patients. Unfortunately, the enrollment of participants in our study was somewhat affected by the outbreak of the Omicron variant of Covid-19, which began in early April 2022 in Taiwan. As a result, a significant proportion of our data points were collected during colder temperatures, as demonstrated in the figure below. Therefore, the interpretation of the temperature impact is limited.

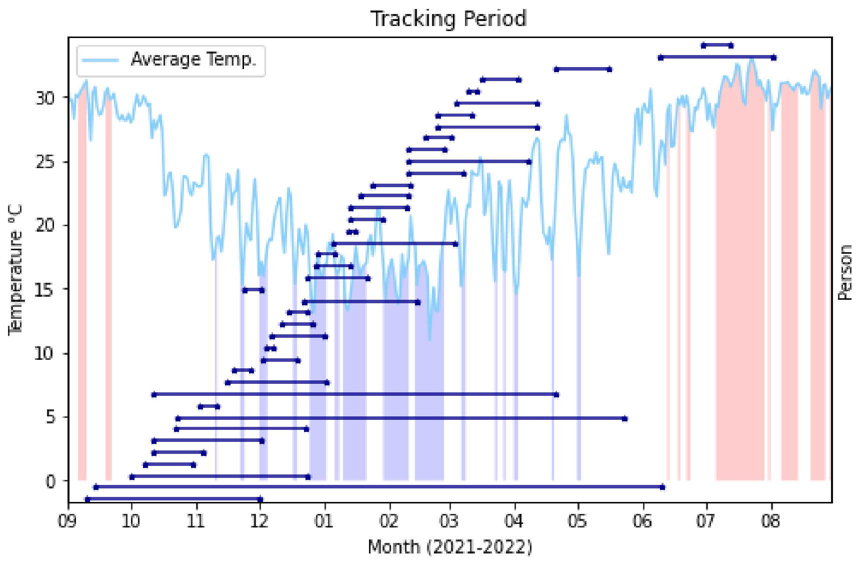

The figure illustrates the temperature distribution during the follow-up period of each case. The X-axis represents the time period from September 2021 to August 2022. Each dark blue horizontal line corresponds to the tracking period of an individual case. The line graph, depicted in sky-blue color, represents the average temperature for each day. The areas filled with red and purple under the curve indicate days with extremely high temperatures (maximum temperature > 35 degrees Celsius) and extremely low temperatures (minimum temperature < 16 degrees Celsius), respectively.

Taking into account the aforementioned limitations, we have made the decision not to incorporate the results into the formal manuscript. We recognize the significance of environmental factors and their potential inclusion in prediction models with a well-designed study and a larger cohort in the future.
